# Benchmarking inflammation-nutrition and TyG-related indices for 5-year mortality risk in adults with questionnaire-defined obstructive sleep apnea: a survey-weighted NHANES derivation cohort with multicenter external validation

**DOI:** 10.1186/s12967-026-08540-0

**Published:** 2026-07-10

**Authors:** Jian Liu, Huiqian Yang, Zheng Wang, Shaban Ahmad, Shuang Zhou, Lingling Zhou, Dan Lou, Zhiqiang Guo, Hui Li, Xiaoming Li

**Affiliations:** 1 Department of Otolaryngology, Shandong Provincial Hospital Affiliated to Shandong First Medical University, Jinan, Shandong 250117 P. R. China; 2 Department of Computer Science, Jamia Millia Islamia, New Delhi, 110025 India; 3 Department of Otolaryngology-Head and Neck Surgery, Shanghai Tenth People’s Hospital, Tongji University School of Medicine, Shanghai, 200000 P. R. China; 4 Department of Otolaryngology-Head and Neck Surgery, Huadong Hospital, Fudan University, Shanghai, 200000 P. R. China; 5 Department of Otolaryngology-Head and Neck Surgery, Shanghai General Hospital (Shanghai First People’s Hospital), Shanghai Jiao Tong University School of Medicine, Shanghai, 200000 P. R. China; 6 Department of Otolaryngology-Head and Neck Surgery, QingPu Branch of Zhongshan Hospital Affiliated to Fudan University, Shanghai, 200000 P. R. China

**Keywords:** Obstructive sleep apnea, Triglyceride-glucose index, Advanced lung cancer inflammation index, Neutrophil percentage-to-albumin ratio, Mortality, Risk prediction

## Abstract

**Background:**

Obstructive sleep apnea (OSA) is associated with excess mortality, and readily obtainable biomarkers may support pragmatic risk stratification in OSA, but their comparative and incremental value remains uncertain. We benchmarked inflammation-nutrition indices and TyG-related metabolic indices for mortality risk in adults with questionnaire-defined OSA and evaluated whether these markers improve 5-year all-cause mortality prediction beyond a basic clinical model.

**Methods:**

We analysed 3,503 adults with questionnaire-defined OSA from the NHANES 2005–2008 and 2015–2018 derivation cohorts. Seven candidate biomarkers were evaluated: TyG, TyG-BMI, TyG-WC, TyG-WHtR, TG/HDL-C, advanced lung cancer inflammation index (ALI), and neutrophil percentage-to-albumin ratio (NPAR). Survey-weighted Cox models and restricted cubic splines were used to characterise mortality associations. Two 5-year all-cause mortality prediction strategies were developed: a tertile-based model (Model 1) and a continuous-scale model (Model 2). Internal validation used bootstrap optimism correction, calibration curves, Brier scores, and decision-curve analysis. Incremental value beyond a prespecified base model was assessed using likelihood-ratio testing, change in AUC, and net reclassification improvement (NRI). External validation was performed in an independent multicenter Chinese cohort of 200 patients from six hospitals. All included patients had at least 5 years of observation time from baseline assessment, allowing complete ascertainment of the binary 5-year all-cause mortality endpoint. The externally validated object was the final Base + Combine model, and its performance was assessed alongside the base model using ROC analysis, calibration plots, calibration intercept and slope, Brier score, and decision-curve analysis.

**Results:**

During a median follow-up of 57.0 months (IQR 33.0–150.0), 293 all-cause deaths occurred in the derivation cohort. Among individual biomarkers, ALI showed the most robust mortality-related signal across analyses, whereas NPAR showed signal in selected single-marker and nonlinear analyses but was less consistent after full multivariable adjustment. TyG-related indices were also variably associated with mortality and contributed mainly within the combined prediction model. In Model 1, the full 7-marker composite model achieved an AUC of 0.735, a bootstrap-corrected AUC of 0.721, and a Brier score of 0.039. In Model 2, the best-performing combined model incorporated TyG-BMI, TyG-WC, TyG-WHtR, TG/HDL-C, and ALI, yielding an AUC of 0.765, a bootstrap-corrected AUC of 0.761, and a Brier score of 0.038. Internal calibration was acceptable for both derivation models, with Model 2 performing better. Decision-curve analysis showed positive net benefit over treat-all and treat-none strategies, with a wider clinically useful threshold range for Model 2. Compared with the base model, the combined biomarker model improved model fit (likelihood-ratio test *p* < 0.001) and reclassification (NRI *p* < 0.001), although the increase in AUC was modest. In the external validation cohort, the prespecified final Base + Combine model achieved an AUC of 0.697 (95% CI 0.581–0.813), compared with 0.672 (95% CI 0.553–0.791) for the base model. External calibration remained imperfect for both models. The base model showed a calibration intercept of −1.609, a calibration slope of 0.385, and a Brier score of 0.138, whereas the final Base + Combine model showed a calibration intercept of 3.782, a calibration slope of 0.402, and a Brier score of 0.089. In external decision-curve analysis, the base model provided greater net benefit at lower threshold probabilities, whereas the final Base + Combine model showed greater net benefit mainly within a narrower higher-threshold range (approximately 0.14–0.30). Given the limited number of external events, these findings should be interpreted as preliminary evidence of transportability rather than as definitive support for a clearly superior prediction tool.

**Conclusions:**

In adults with questionnaire-defined OSA from NHANES, inflammation-nutrition markers, particularly ALI, showed stronger mortality-related signal than TyG-related indices at the single-marker level, while NPAR showed supportive signal in selected analyses. More importantly, a combined inflammatory-metabolic biomarker model provided modest incremental enrichment for 5-year all-cause mortality risk benchmarking beyond routine clinical variables. In an external multicenter cohort with PSG-confirmed OSA, the final Base + Combine model showed moderate but preliminary transportability, with slightly improved discrimination but poor calibration. Any incremental net benefit in external decision-curve analysis appeared to be confined to a relatively narrow higher-threshold range. These findings support pragmatic risk benchmarking rather than immediate use as a clearly superior clinical prediction tool.

**Supplementary information:**

The online version contains supplementary material available at 10.1186/s12967-026-08540-0.

## Introduction

Obstructive sleep apnea (OSA) affects approximately 1 billion adults worldwide and is associated with excess all-cause and cardiovascular mortality [1–3]. Intermittent hypoxia, sleep fragmentation, sympathetic activation, oxidative stress, and chronic low-grade inflammation are all implicated in the adverse cardiometabolic consequences of OSA [4–7]. However, risk stratification in routine practice remains limited. Polysomnography-derived measures (PSG), including apnea-hypopnea index (AHI) and hypoxic burden, provide important physiological information, but these metrics are not always available in broad epidemiologic or low-resource clinical settings [8–10]. In such contexts, readily obtainable biomarkers may offer a pragmatic complement for identifying individuals at higher risk [11].

In recent years, the triglyceride-glucose (TyG) index and its obesity-related derivatives (TyG-BMI, TyG-WC, TyG-WHtR, and TG/HDL-C ratio) have emerged as inexpensive surrogate markers of insulin resistance and metabolic dysfunction, with prognostic relevance across several cardiometabolic settings [12–19]. In parallel, inflammation-nutrition indices such as ALI and NPAR integrate inflammatory and nutritional information from routine blood tests and anthropometry and have emerged as potentially informative markers in chronic disease populations [20–24]. Because OSA sits at the intersection of adiposity, insulin resistance, systemic inflammation, and cardiopulmonary risk, comparing these candidate biomarker families head-to-head is clinically relevant.

Recent translational evidence further supports the systemic nature of OSA beyond sleep-disordered breathing itself. Repetitive hypoxemia in OSA has been associated with endothelial glycocalyx perturbation, oxidative-inflammatory pathway activation, and immunothrombotic endothelial dysfunction. In addition, OSA has been linked to reduced muscle quality, increased fat infiltration, and metabolic abnormalities. These findings suggest that OSA-related risk may involve inflammatory, endothelial, nutritional, metabolic, and body-composition pathways, providing a rationale for evaluating routinely available biomarker indices in this population [25, 26].

That said, a translationally useful paper in this area must do more than rank biomarkers by association strength. The more clinically relevant question is whether routine biomarkers can improve risk stratification beyond basic clinical information and whether such performance is reproducible outside the derivation dataset. This distinction is particularly important in secondary NHANES analyses, where OSA is questionnaire-defined rather than objectively phenotyped and where associations alone do not establish decision-support utility.

In the present study, we therefore reframed the analysis around clinically grounded risk prediction and pragmatic risk benchmarking. Specifically, we benchmarked seven routinely available inflammatory and metabolic indices in adults with probable OSA from NHANES, developed a pragmatic 5-year all-cause mortality risk model, quantified calibration and clinical utility, tested incremental value beyond a prespecified base model, and externally evaluated the final Base + Combine model in an independent multicenter cohort with PSG-confirmed OSA [27–29]. Importantly, this design deliberately bridged a questionnaire-defined, population-based derivation setting and a PSG-confirmed, clinic-based validation setting, thereby providing a more stringent and scientifically informative test of transportability. We treated respiratory mortality analyses as supportive exploratory analyses given the sparse number of respiratory deaths in the derivation dataset.

## Methods

### Study design and data source

This study comprised a derivation analysis based on NHANES and an external validation analysis based on an independent multicenter Chinese cohort. The derivation cohort included adults with questionnaire-defined OSA from NHANES 2005–2008 and 2015–2018. Mortality follow-up was obtained through linkage to the National Death Index through December 31, 2019. The external validation cohort included 200 adults with polysomnography-confirmed OSA from six hospitals in China after exclusion of individuals with incomplete key variables required for model evaluation. All included patients had at least 5 years of observation time from baseline assessment. OSA in the validation cohort was defined objectively as an apnea-hypopnea index (AHI) ≥5 events/hour [27, 28].

In NHANES, OSA was defined using symptom-based questionnaire items, consistent with prior secondary analyses in this field. Specifically, participants were classified as having probable OSA if they reported frequent snoring, frequent snorting/gasping or witnessed apneas, or excessive daytime sleepiness despite apparently adequate habitual sleep duration. Because this definition is questionnaire-based rather than polysomnography-based, the derivation cohort is best interpreted as a probable-OSA population, and the resulting models should be viewed accordingly.

For external validation, an independent cohort was assembled from six hospitals in China. All patients in the validation cohort underwent overnight polysomnography, and OSA was defined objectively as an apnea-hypopnea index (AHI) ≥5 events/hour [27, 28]. After exclusion of individuals with missing key variables required for model evaluation, 200 patients were included in the final external validation analysis. Baseline data had been recorded before outcome ascertainment, and 5-year vital status was subsequently determined for all included patients, allowing complete ascertainment of the binary 5-year all-cause mortality endpoint. All six participating centers were high-level tertiary hospitals in China. For the external validation cohort, blood samples were obtained after overnight fasting and biomarker measurements were performed according to a harmonized protocol across centers. Triglycerides, glucose, HDL-C, albumin, and leukocyte differentials were measured under standardized clinical laboratory procedures, and all derived indices, including TyG, TyG-BMI, TyG-WC, TyG-WHtR, TG/HDL-C, ALI, and NPAR, were calculated using the same prespecified formulas. These procedures were implemented to improve inter-center laboratory comparability in the external validation setting.

### Study population and covariates

In the NHANES derivation cohort, we began with adults who met the study definition of OSA and excluded individuals with missing biomarker data, missing key covariates, or pregnancy, yielding a final analytic sample of 3,503 participants. The extent of attrition was substantial and is acknowledged as a limitation because it may affect generalizability. Candidate covariates were prespecified on clinical grounds. The base clinical framework included age, sex, body mass index (BMI), hypertension, diabetes, smoking, and alcohol consumption. Additional descriptive covariates used in the etiologic models included race/ethnicity, education, poverty-income ratio, and marital status. Sex, diabetes, and hypertension were coded as binary variables; age and smoking status were coded as 3-level categorical variables; BMI was coded as a 4-level categorical variable; and alcohol consumption was coded as a 5-level categorical variable. The reference categories were age < 45 years, male sex, normal BMI, no diabetes, no hypertension, never smoking, and never drinking.

### Biomarkers, outcomes, and exploratory endpoints

Seven biomarkers were examined: TyG, TyG-BMI, TyG-WC, TyG-WHtR, TG/HDL-C, ALI, and NPAR. The primary endpoint for the prediction analyses was 5-year all-cause mortality. Prevalent cardiovascular disease (CVD), cardiovascular mortality, and respiratory disease (RD) mortality were retained as supportive analyses. Because only 13 respiratory deaths occurred in the derivation cohort, RD mortality and all sex-stratified mortality analyses were treated as exploratory.

### Statistical analysis for association benchmarking

All NHANES analyses accounted for the complex survey design. Survey-weighted logistic regression was used for prevalent CVD and survey-weighted Cox proportional hazards models were used for all-cause, CVD, and RD mortality. The proportional hazards assumption for the Cox models was assessed using Schoenfeld residuals. No material violation was detected for the main all-cause mortality models. Restricted cubic splines were used to examine potential nonlinear relationships with all-cause mortality. For the main association analyses, emphasis was placed on effect sizes, confidence intervals, and consistency across outcomes rather than on biomarker ranking language. Given the questionnaire-defined OSA framework and residual confounding concerns, results were interpreted as benchmark comparisons within a secondary epidemiologic dataset rather than as evidence of biological dominance.

### Prediction model development, internal validation, and clinical utility

Two 5-year all-cause mortality prediction approaches were developed. Model 1 used tertile-based representations of the seven biomarkers. Model 2 modelled the biomarkers on the continuous scale, with transformations or rescaling applied where needed to improve stability and interpretability. For Model 2, candidate continuous biomarker combinations were compared according to apparent AUC, bootstrap-corrected AUC, calibration performance, Brier score, decision-curve characteristics, and clinical interpretability. Collinearity among biomarkers included in the final continuous-scale model was assessed using pairwise Spearman correlation analysis and variance inflation factors (VIFs). A Spearman correlation coefficient greater than 0.70 was considered to indicate strong correlation, and a VIF greater than 5 was considered to indicate potentially relevant multicollinearity. Internal validation was performed using bootstrap optimism correction. For each model, apparent AUC and optimism-corrected AUC were reported. Calibration was examined using bootstrap-corrected calibration curves, and prediction error was summarised with the Brier score [30]. Clinical utility was evaluated using decision curve analysis (DCA) by comparing model-based strategies with treat-all and treat-none strategies [31, 32]. To assess incremental value beyond routine clinical information, we constructed a prespecified base model including age, sex, BMI, hypertension, diabetes, smoking, and alcohol consumption. We then sequentially added ALI, NPAR, and the selected combined biomarker panel. Improvement was evaluated with the likelihood-ratio test, change in AUC, and net reclassification improvement (NRI). The exact composite-model formulas were reported to improve reproducibility. The exact linear predictors of the base model and the final Base + Combine model were reported to improve reproducibility. The full regression coefficients for both models are provided in Supplementary Table 1. The exact linear predictor formulas of the base clinical model and the final Base + Combine model, together with a worked example of risk calculation, are provided in the Supplementary Methods to improve reproducibility and usability. In the final combined model, TyGBMI100 and TyGWC100 denote TyG-BMI and TyG-WC, respectively, divided by 100, whereas TG/HDL-C and ALI were entered on the natural logarithmic scale. For both models, the relative hazard was calculated as HR = exp(η).

### External validation

The prespecified final Base + Combine model from the derivation stage was applied to the independent multicenter external validation cohort without refitting. To align the derivation and validation frameworks, the performance of the base model was also evaluated in the same cohort. To improve laboratory comparability across the six Chinese centers, all biomarker measurements in the external cohort were obtained from fasting samples and processed according to a harmonized protocol, with derived indices calculated using identical prespecified formulas. All included patients in the external validation cohort had at least 5 years of observation time from baseline assessment. Baseline data had been recorded before outcome ascertainment, and 5-year vital status was subsequently determined for all included patients, allowing complete ascertainment of the binary 5-year all-cause mortality endpoint. External discrimination was summarised using ROC analysis with 95% confidence intervals. Calibration was assessed using grouped calibration plots, calibration intercept and slope, and the Brier score. External decision-curve analysis was additionally performed to compare the clinical utility of the base model and the final Base + Combine model against treat-all and treat-none strategies.

### General statistical procedures

Baseline characteristics were compared according to study outcomes, including prevalent CVD, all-cause mortality, cardiovascular mortality, and RD mortality. Continuous variables were assessed for normality using the Shapiro–Wilk test. Normally distributed variables are presented as mean ± standard deviation (SD) and were compared using the independent-samples t test; non-normally distributed variables are presented as median (interquartile range [IQR]) and were compared using the Mann–Whitney U test. Categorical variables are presented as counts and percentages and were compared using the chi-square test. Baseline comparisons in the derivation cohort were performed in both unweighted and survey-weighted forms. All statistical tests were two-sided, and *p* values < 0.05 were considered statistically significant. Statistical analyses were performed using R software version 4.4.3.

## Results

### Participant characteristics

**Fig. 1:**
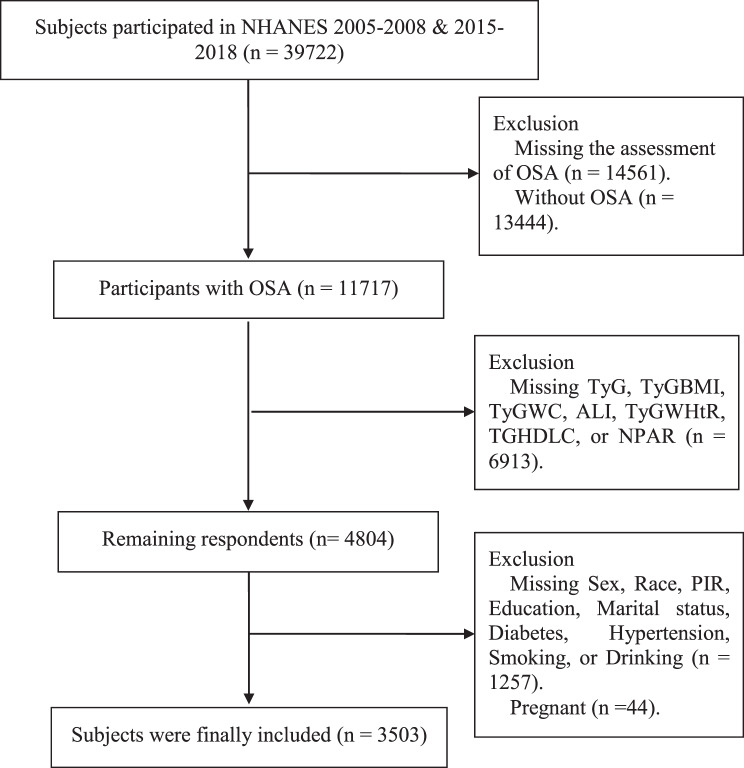
Study flowchart for derivation cohort selection from NHANES

A total of 3,503 adults with questionnaire-defined OSA were included in the derivation cohort (Fig. 1), of whom 415 (11.85%) had prevalent CVD. During a median follow-up of 57.0 months (IQR, 33.0–150.0), 293 participants (8.36%) died from any cause, including 82 cardiovascular deaths (2.34%) and 13 respiratory disease (RD) deaths (0.37%). Detailed unweighted and survey-weighted baseline characteristics, stratified by prevalent CVD and mortality outcomes, are presented in Supplementary Tables 2 and 3. Overall, baseline differences were more evident for prevalent CVD and all-cause mortality than for RD mortality, due to the small number of events, which limited interpretability. Accordingly, RD-related analyses were treated as supportive exploratory analyses rather than emphasised as primary findings.

The selection process for the external validation cohort is shown in Supplementary Figure 1. The external validation cohort included 200 adults with PSG-confirmed OSA recruited from six hospitals in China. All included patients had at least 5 years of observation time from baseline assessment, allowing complete determination of 5-year all-cause mortality status for the entire cohort. At the 5-year time point, 182 patients were alive and 18 had died. OSA in the validation cohort was defined objectively as an apnea-hypopnea index (AHI) ≥5 events/hour. Baseline characteristics of the derivation and validation cohorts are compared in Supplementary Table 4. In brief, the validation cohort differed from the derivation cohort in several demographic and clinical characteristics, supporting a deliberately heterogeneous and therefore scientifically informative external evaluation of model transportability across distinct epidemiologic and clinical settings. A descriptive center-level summary of sample counts, deaths, and key biomarker distributions in the external validation cohort is provided in Supplementary Table 8.

### Benchmarking of individual biomarkers

In the derivation cohort, ALI and NPAR yielded the clearest and most consistent mortality-related signals across analyses, whereas TyG-related indices were less stable after multivariable adjustment. For prevalent CVD, only ALI showed a significant association in the fully adjusted model, with tertile 2 associated with lower odds relative to tertile 1 (OR, 0.629; 95% CI, 0.413–0.959; *p* = 0.032) (Supplementary Table 5). No other biomarker remained significantly associated with prevalent CVD after full adjustment.

For all-cause mortality, higher ALI was consistently associated with lower risk, with significant associations for tertile 2 versus tertile 1 (HR, 0.563; 95% CI, 0.413–0.769; *p* < 0.001) and tertile 3 versus tertile 1 (HR, 0.607; 95% CI, 0.407–0.906; *p* = 0.015) in the fully adjusted model (Table 1). Selected TyG-derived adiposity indices also showed inverse associations, including TyG-BMI tertile 2 (HR, 0.616; 95% CI, 0.415–0.913; *p* = 0.016), TyG-BMI tertile 3 (HR, 0.570; 95% CI, 0.405–0.802; *p* = 0.001), TyG-WC tertile 3 (HR, 0.683; 95% CI, 0.486–0.960; *p* = 0.028), and TyG tertile 3 (HR, 0.664; 95% CI, 0.445–0.990; *p* = 0.045), whereas NPAR was not significant after full adjustment (Table 1).

**Table 1:** HR (95% CIs) of all-cause mortality according to tertiles of seven biomarkers among OSA in NHANES

Biomarkers	HR (95% CI), *p* value		
	Model 1	Model 2	Model 3
TyG			
T1 (6.558 – <8.406)	Reference	Reference	Reference
T2 (8.406 – <8.955)	1.048 (0.711, 1.543), 0.813	0.799 (0.563, 1.132), 0.207	0.742 (0.521, 1.056), 0.098
T3 (8.955–12.444)	1.312 (0.894, 1.927), 0.165	0.937 (0.656, 1.337), 0.718	0.664 (0.445, 0.990), 0.045
TyG-BMI			
T1 (125.8 – <232.4)	Reference	Reference	Reference
T2 (232.4 – <287.7)	0.791 (0.536, 1.167), 0.237	0.656 (0.453, 0.951), 0.026	0.616 (0.415, 0.913), 0.016
T3 (287.7–620.9)	0.894 (0.625, 1.278), 0.538	0.776 (0.545, 1.106), 0.161	0.570 (0.405, 0.802), 0.001
TyG-WC			
T1 (493.0 – <822.9)	Reference	Reference	Reference
T2 (822.9 – <969.5)	1.301 (0.939, 1.803), 0.113	0.959 (0.692, 1.331), 0.803	0.832 (0.577, 1.200), 0.325
T3 (969.5–1697.4)	1.383 (0.993, 1.927), 0.055	0.972 (0.701, 1.348), 0.865	0.683 (0.486, 0.960), 0.028
TyG-WHtR			
T1 (2.763 – <4.891)	Reference	Reference	Reference
T2 (4.891 – <5.762)	1.169 (0.821, 1.663), 0.386	0.819 (0.580, 1.158), 0.259	0.697 (0.475, 1.023), 0.065
T3 (5.762–10.229)	1.666 (1.115, 2.490), 0.013	1.097 (0.752, 1.599), 0.631	0.735 (0.499, 1.083), 0.119
TG/HDL-C			
T1 (0.070 – <0.687)	Reference	Reference	Reference
T2 (0.687 – <1.318)	0.809 (0.547, 1.196), 0.288	0.785 (0.542, 1.136), 0.199	0.750 (0.501, 1.123), 0.163
T3 (1.318–40.403)	0.932 (0.645, 1.347), 0.708	0.926 (0.669, 1.282), 0.644	0.782 (0.568, 1.076), 0.131
ALI			
T1 (4.625 – <54.159)	Reference	Reference	Reference
T2 (54.159 – <79.330)	0.456 (0.324, 0.641), <0.001	0.560 (0.403, 0.779), <0.001	0.563 (0.413, 0.769), <0.001
T3 (79.330–2814.32)	0.451 (0.315, 0.645), <0.001	0.513 (0.351, 0.750), <0.001	0.607 (0.407, 0.906), 0.015
NPAR			
T1 (85.7 – <1273.7)	Reference	Reference	Reference
T2 (1273.7 – <1488.6)	1.020 (0.661, 1.575), 0.929	0.906 (0.569, 1.444), 0.678	0.772 (0.468, 1.271), 0.308
T3 (1488.6–2491.9)	2.140 (1.459, 3.140), <0.001	1.814 (1.217, 2.757), 0.004	1.402 (0.936, 2.100), 0.101

HR, Hazard ratio; 95% CI, 95% confidence interval.

Model 1 was not adjusted for confounding factors;

Model 2 was adjusted as age, and race;

Model 3 was adjusted as model 2 plus family poverty income ratio, marital status, education, drinking status, smoking status, diabetes, and hypertension

For cardiovascular mortality, ALI remained the only biomarker with a significant association after full adjustment, with tertile 3 associated with a lower risk than tertile 1 (HR, 0.378; 95% CI, 0.182–0.782; *p* = 0.009) (Supplementary Table 6). No other biomarker was significantly associated with cardiovascular mortality after full adjustment.

For RD mortality, which was treated as exploratory because of the small number of events, ALI tertile 2 was associated with lower risk (HR, 0.052; 95% CI, 0.007–0.384; *p* = 0.004), whereas NPAR tertile 3 was associated with higher risk (HR, 8.896; 95% CI, 1.072–73.790; *p* = 0.043) in the parsimoniously adjusted model (Supplementary Table 7). These findings should be interpreted cautiously, given the sparse number of respiratory deaths.

Taken together, these results support a cautious benchmarking interpretation: in this specific secondary NHANES dataset, inflammation–nutrition indices appeared more informative than insulin-resistance surrogates, although these differences should not be interpreted as evidence of biological dominance. Possible alternative explanations include general illness burden, nutritional reserve, obesity-paradox effects, residual confounding, and symptom-based OSA misclassification.

### Nonlinear associations with all-cause mortality

**Fig. 2:**
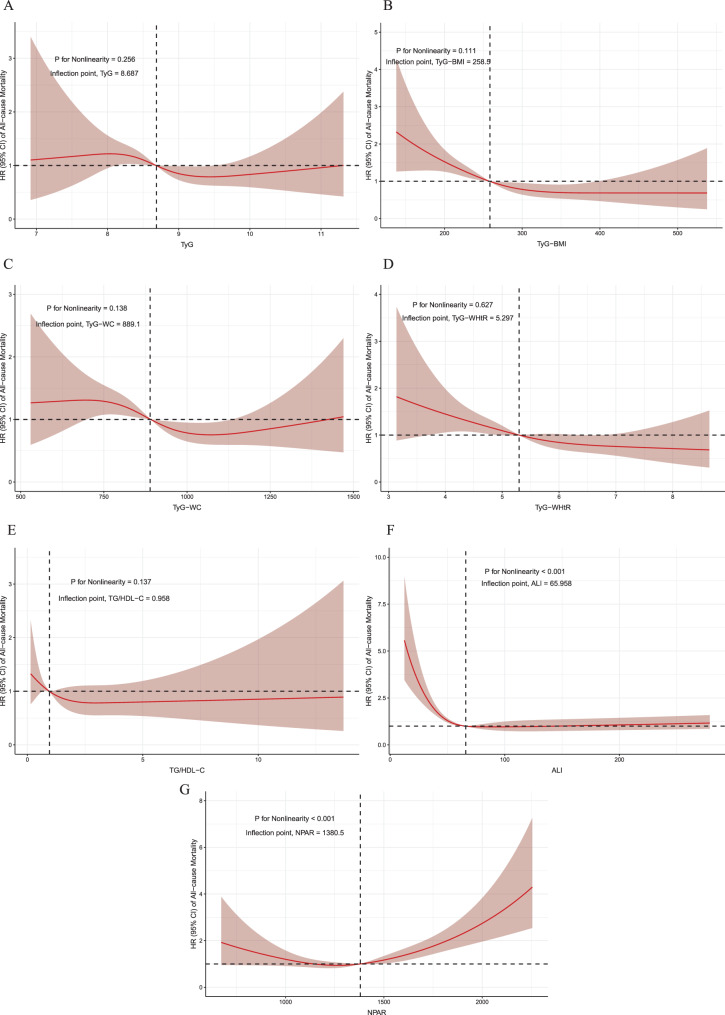
Restricted cubic spline analyses of associations between biomarker levels and all-cause mortality in adults with questionnaire-defined OSA. Restricted cubic spline curves showing the multivariable-adjusted associations of (**A**) TyG, (**B**) TyG-BMI, (**C**) TyG-WC, (**D**) TyG-WHtR, (**E**) TG/HDL-C, (**F**) ALI, and (**G**) NPAR with all-cause mortality. Red solid lines indicate estimated hazard ratios, and shaded areas indicate 95% confidence intervals. Horizontal dashed lines indicate HR = 1.0, and vertical dashed lines indicate the estimated inflection points

Restricted cubic spline analyses revealed significant nonlinear relationships between ALI and NPAR and all-cause mortality. For ALI, risk increased as ALI decreased, with an apparent threshold around 65.958, below which lower ALI was associated with higher mortality risk and above which the curve largely plateaued (Fig. 2F). For NPAR, an inflection point was observed at 1380.5, above which mortality risk increased progressively with higher NPAR values, whereas below this level, no clear gradient was evident (Fig. 2G). No statistically significant nonlinearity was observed for TyG, TyG-WC, TyG-WHtR, or TG/HDL-C, although TyG-BMI did not reach formal statistical significance for nonlinearity, the spline curve suggested a possible threshold-type pattern (Fig. 2A–E).

### Development of 5-year all-cause mortality prediction models

**Fig. 3:**
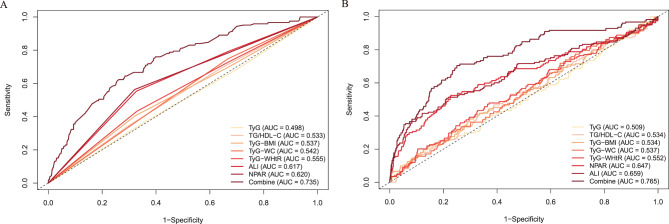
Discriminative performance of individual biomarkers and combined models for 5-year all-cause mortality prediction. Receiver operating characteristic (ROC) curves for the prediction of 5-year all-cause mortality using seven candidate biomarkers and the combined model. (**A**) Model 1, in which biomarkers were categorised into tertiles. (**B**) Model 2, in which biomarkers were modelled on the continuous scale. In both approaches, the combined model showed the highest discriminative performance compared with any single biomarker

The Kaplan–Meier-estimated 5-year survival rate in the derivation cohort was 95.7% (95% CI, 95.0%–96.5%), corresponding to an estimated 5-year mortality risk of approximately 4.3%. We evaluated the seven biomarkers for predicting 5-year all-cause mortality using two modelling approaches: a tertile-based model (Model 1) and a continuous-scale model (Model 2). In Model 1, NPAR showed the highest discrimination among single biomarkers (AUC, 0.620), followed closely by ALI (AUC, 0.617). The full 7-marker composite model achieved the best overall performance, with an AUC of 0.735, a bootstrap-corrected AUC of 0.721, a Youden index of 0.385, sensitivity of 0.673, specificity of 0.712, and a Brier score of 0.039 (Fig. 3A; Table 2).

**Table 2:** ROC analysis of seven biomarkers, and combined index in predicting 5-year all-cause mortality in adults with OSA in NHANES

Biomarkers	Model 1				Model 2			
	AUC	Youden index	Sen	Spe	AUC	Youden index	Sen	Spe
TyG	0.498	0.028	0.360	0.668	0.509	0.077	0.231	0.846
TyG-BMI	0.537	0.079	0.409	0.670	0.534	0.086	0.401	0.685
TyG-WC	0.542	0.094	0.757	0.337	0.537	0.103	0.724	0.379
TyG-WHtR	0.555	0.093	0.423	0.670	0.552	0.101	0.481	0.620
TG/HDL-C	0.533	0.046	0.711	0.335	0.534	0.072	0.438	0.634
ALI	0.617	0.219	0.543	0.686	0.659	0.286	0.500	0.786
NPAR	0.620	0.247	0.569	0.678	0.647	0.300	0.534	0.766
Combine	0.735	0.385	0.673	0.712	0.765	0.479	0.723	0.756

AUC, area under the curve; Sen, sensitivity; Spe, specificity

Model 1, TyG, TyG-BMI, TyG-WC, TyG-WHtR, TG/HDL-C, ALI, and NPAR grouped by tertiles

Model 2, TyG, TyG-BMI, TyG-WC, TyG-WHtR, TG/HDL-C, ALI, and NPAR as continuous variables

In Model 2, ALI was the most informative single biomarker (AUC, 0.659), followed by NPAR (AUC, 0.647). Several TyG-related indices showed limited standalone discriminative ability and should not be interpreted as useful individual predictors. After performance-based variable selection, the best-performing combined model included TyG-BMI, TyG-WC, TyG-WHtR, TG/HDL-C, and ALI, and achieved an AUC of 0.765, a bootstrap-corrected AUC of 0.761, a Youden index of 0.479, sensitivity of 0.723, specificity of 0.756, and a Brier score of 0.038 (Fig. 3B; Table 2). Pairwise Spearman correlation and VIF analyses showed expected collinearity among TyG-BMI, TyG-WC, and TyG-WHtR, whereas TG/HDL-C and ALI showed lower collinearity (Supplementary Tables 9 and 10). Therefore, the final combined model was interpreted primarily as a prediction model rather than an etiological model. Overall, these results indicate that multimarker combinations outperformed any single biomarker and that the continuous-scale formulation performed better than the tertile-based formulation.

### Calibration and decision-curve analysis

**Fig. 4:**
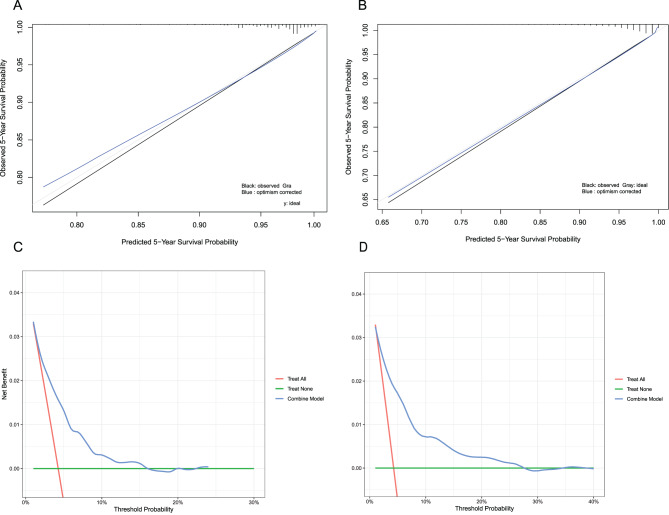
Calibration and decision-curve analysis of the 5-year all-cause mortality prediction models. (**A**) Bootstrap-corrected calibration curve for model 1. (**B**) Bootstrap-corrected calibration curve for model 2. (**C**) Decision-curve analysis for model 1. (**D**) Decision-curve analysis for model 2

Bootstrap-corrected calibration curves demonstrated acceptable agreement between predicted and observed 5-year survival probabilities for both prediction models, with visually better calibration for Model 2 than for Model 1 (Fig. 4A, B). Decision-curve analysis further supported the clinical usefulness of these models. Both models showed greater net benefit than the treat-all and treat-none strategies over clinically relevant threshold probabilities, although Model 2 had a wider useful range. Specifically, Model 1 showed a positive net benefit over a threshold range of 0–16% (Fig. 4C), whereas Model 2 remained beneficial up to an approximate threshold of 27% (Fig. 4D). These findings supported selecting Model 2 as the final prediction framework.

### Incremental value beyond the base model

To determine whether the biomarkers added value beyond routine clinical information, we compared a prespecified base model against three augmented models: Base + ALI, Base + NPAR, and Base + Combine (Fig. 5). All three augmented models significantly improved model fit relative to the base model on likelihood-ratio testing (all *p* < 0.001) (Table 3). To improve quantitative transparency, Table 3 now reports not only the *p* values for likelihood-ratio testing, AUC comparison, and NRI, but also the actual effect sizes, including ΔAUC with 95% confidence intervals and category-free NRI estimates with 95% confidence intervals. However, gains in discrimination were modest: AUC increased from 0.817 for the base model to 0.825 for Base + ALI, 0.830 for Base + NPAR, and 0.835 for Base + Combine. The Youden index was 0.537 for the base model, 0.544 for Base + ALI, 0.578 for Base + NPAR, and 0.576 for Base + Combine. Sensitivity/specificity were 0.870/0.667 for the base model, 0.725/0.819 for Base + ALI, 0.836/0.742 for Base + NPAR, and 0.810/0.766 for Base + Combine. These AUC differences were directionally favourable but not statistically significant in the AUC comparisons (*p* = 0.561, 0.133, and 0.108, respectively). In contrast, reclassification analysis showed a significant improvement for the combined biomarker model versus the base model (NRI *p* < 0.001), whereas the corresponding NRI differences for Base + ALI and Base + NPAR were not statistically significant (*p* = 0.557 and 0.617, respectively) (Table 3). Taken together, these results suggest that the combined biomarker panel provided modest incremental enrichment beyond routine clinical variables, although the gain in overall discrimination was limited and not statistically significant in AUC-based comparisons. Full regression coefficients of the base model and the final Base + Combine model are presented in Supplementary Table 1. In brief, older age, hypertension, diabetes, and smoking contributed to higher predicted 5-year mortality risk, whereas female sex and overweight/obesity were associated with lower predicted risk relative to the reference categories. In the final Base + Combine model, lower TyG-BMI and lower ALI contributed most strongly to higher predicted risk, while the biomarker panel as a whole improved risk estimation beyond the base clinical framework.

**Table 3:** Comparative predictive performance of four models for 5-year all-cause mortality in adults with OSA in NHANES

Model	AUC	ΔAUC vs. base (95%CI)	Youden index	Sen	Spe	*p* value #			Category-free NRI vs. base (95%CI)
						likelihood-ratio test	AUC	NRI	
Base	0.817	Reference	0.537	0.870	0.667	NA	NA	NA	Reference
Base + ALI	0.825	0.007 (−0.016, 0.026)	0.544	0.725	0.819	<0.001	0.561	0.557	0.033 (−0.058, 0.144)
Base + NPAR	0.830	0.013 (−0.005, 0.030)	0.578	0.836	0.742	<0.001	0.133	0.617	0.033 (−0.055, 0.140)
Base + Combine	0.835	0.018 (−0.007, 0.039)	0.576	0.810	0.766	<0.001	0.108	<0.001	0.277 (0.143, 0.352)

AUC, area under the curve; Sen, sensitivity; Spe, specificity

#, Base + ALI, Base + NPAR, or Base + Combine vs. Base

**Fig. 5:**
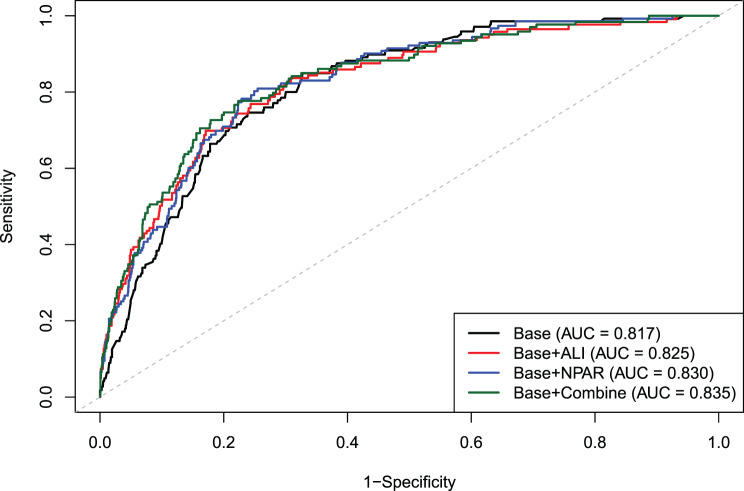
ROC comparison of the base model and biomarker-augmented models for 5-year all-cause mortality prediction. Receiver operating characteristic curves comparing the base model with three augmented models: base + ALI, Base + NPAR, and Base + Combine. The combined biomarker model showed the highest AUC, although the absolute improvement in discrimination over the base model was modest

### External validation of the final Base + Combine model

**Fig. 6:**
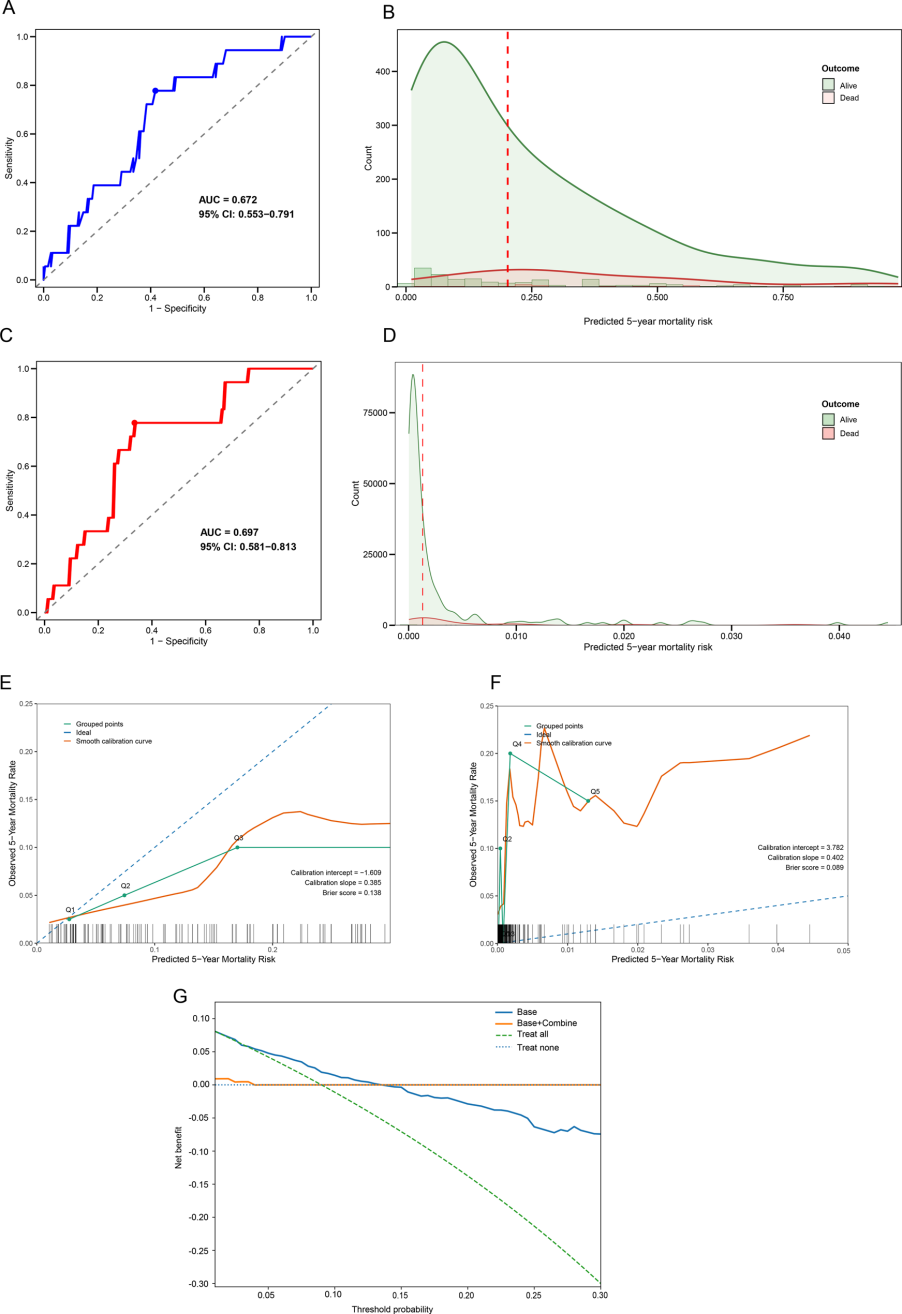
External validation of the base model and the final Base + Combine model in the independent multicenter cohort with PSG-confirmed OSA. (**A**) Receiver operating characteristic (ROC) curve of the base model for 5-year all-cause mortality prediction in the external validation cohort. (**B**) Distribution of predicted 5-year mortality risk by outcome group for the base model. (**C**) ROC curve of the final Base + Combine model in the external validation cohort. (**D**) Distribution of predicted 5-year mortality risk by outcome group for the final Base + Combine model. (**E**) External calibration plot of the base model. (**F**) External calibration plot of the final Base + Combine model. (**G**) External decision-curve analysis comparing the net benefit of the base model and the final Base + Combine model

The external validation cohort consisted of 200 adults with PSG-confirmed OSA recruited from six hospitals in China. All included patients had at least 5 years of observation time from baseline assessment, allowing complete determination of the binary 5-year all-cause mortality endpoint for the entire cohort. At the 5-year time point, 182 patients were alive and 18 had died. OSA was defined objectively as an apnea-hypopnea index (AHI) ≥5 events/hour. In external ROC analysis, the base model achieved an AUC of 0.672 (95% CI 0.553–0.791), whereas the prespecified final Base + Combine model achieved an AUC of 0.697 (95% CI 0.581–0.813) (Fig. 6A, C). The predicted-risk distributions showed some separation between survivors and non-survivors for both models, although substantial overlap remained, particularly for the final Base + Combine model because most predicted risks were concentrated at the lower end of the distribution (Fig. 6B, D).

External calibration analyses indicated imperfect calibration for both models. For the base model, the calibration intercept was −1.609, the calibration slope was 0.385, and the Brier score was 0.138 (Fig. 6E). For the final Base + Combine model, the calibration intercept was 3.782, the calibration slope was 0.402, and the Brier score was 0.089 (Fig. 6F). These findings suggest that, although the final Base + Combine model showed slightly better discrimination and lower overall prediction error, absolute risk calibration remained suboptimal in the external cohort.

External decision-curve analysis further showed that the clinical utility of biomarker augmentation was threshold-dependent rather than uniform. The base model showed greater net benefit across lower threshold probabilities, whereas the final Base + Combine model provided greater net benefit mainly at higher threshold probabilities (approximately 0.14–0.30) (Fig. 6G). Because the observed 5-year mortality in the external cohort was 9%, this pattern suggests that the practical external usefulness of the combined model may be narrower than implied by discrimination alone. Given the limited number of external deaths, these findings should be interpreted as preliminary rather than definitive. This external test is scientifically informative because it bridges a questionnaire-defined, population-based derivation cohort and a PSG-confirmed, clinic-based validation cohort, rather than merely repeating validation in a closely matched sample. Overall, the external validation should be interpreted as exploratory evidence of transportability only. The final Base + Combine model should not be considered clinically applicable for direct absolute risk prediction or decision-making in PSG-confirmed OSA populations without further recalibration and validation. Given the multicenter nature of the validation cohort and the limited number of external events, Supplementary Table 8 provides a descriptive center-level summary to improve transparency regarding cohort composition across sites.

### Exploratory analyses

Only 13 respiratory deaths occurred in the derivation cohort; therefore, RD mortality results were retained only as supportive exploratory observations. In this context, ALI showed an inverse association with RD mortality, whereas NPAR showed a positive association in exploratory analyses. Given the sparse number of respiratory deaths, these findings should be regarded as preliminary (Supplementary Figures 2–8).

## Discussion

PSG remains the reference standard for OSA [27]. However, PSG-based assessment is not always readily available in primary hospitals, community settings, or low-resource clinics, and there remains a lack of simple, scalable, and widely generalisable approaches for risk stratification and mortality prediction in routine practice [33]. In this context, the present analysis shows that, within a secondary NHANES dataset of adults with questionnaire-defined OSA, inflammation-nutrition indices, particularly ALI, carried stronger and more consistent mortality-related signals than TyG-related insulin-resistance surrogates, while NPAR showed supportive signal in selected analyses but was less robust after full multivariable adjustment.

From a translational perspective, recent studies have highlighted the potential clinical relevance of targeting metabolic vulnerability in OSA. GLP-1 receptor agonists have been systematically evaluated in patients with OSA complicated by obesity or type 2 diabetes, and combined vitamin D3 plus SGLT2 inhibitor therapy has shown potential benefits for cardiometabolic outcomes and quality of life in hypertensive obese OSA patients [34, 35]. These findings are consistent with our interpretation that inflammation-nutrition and metabolic indices may capture clinically relevant systemic vulnerability in OSA, although they should be considered complementary to, rather than substitutes for, PSG-derived physiological measures.

Several OSA prognostic approaches have been reported previously. For example, models derived from the Sleep Heart Health Study and other sleep-cohort datasets have shown that long-term mortality risk in OSA can be predicted using multidimensional physiological and clinical information, including PSG-derived sleep variables, hypoxic burden, heart-rate variability features, and machine-learning-based risk profiling [8, 36, 37]. These studies collectively suggest that OSA prognosis is better captured by integrated physiological and clinical risk assessment than by the apnea-hypopnea index alone.

However, most published OSA prognostic approaches require PSG-derived physiological signals, heart-rate variability features, or complex machine-learning frameworks, which limits their direct applicability in large epidemiologic datasets, community screening settings, or lower-resource clinical scenarios where PSG-derived severity metrics are unavailable. A direct head-to-head comparison with these models was therefore not feasible in the present study because their target outcomes, prediction horizons, modelling frameworks, and required input variables differed from those of our 5-year all-cause mortality model. Accordingly, the present biomarker-based model should be viewed as a pragmatic risk-benchmarking complement to, rather than a replacement for, established PSG-based prognostic models. Its main potential value lies in using routinely available inflammatory, nutritional, and metabolic markers to provide modest incremental enrichment when sleep-study-derived severity metrics are not available [38–40].

Among the candidate biomarkers, ALI was the most robust single-marker predictor on the continuous scale. NPAR showed supportive signal in selected analyses, but its association with mortality was attenuated after full multivariable adjustment. These inflammation-nutrition markers are attractive because they combine routinely available laboratory and anthropometric information and can be calculated at low cost. Their apparent value may reflect their ability to capture multiple pathophysiologic dimensions simultaneously, including systemic inflammation, nutritional reserve, and overall illness burden. By contrast, TyG-related indices mainly reflect metabolic and adiposity-related risk. Although several TyG-related indices showed limited standalone discrimination and should not be used as individual prediction markers, their inclusion in the candidate set was prespecified because they capture metabolic-adiposity dimensions that are biologically relevant to OSA-related risk. Their retention in the final combined model should therefore be interpreted cautiously as evidence of possible complementary information when integrated with inflammatory-nutritional markers, rather than as evidence of independent predictive utility [41]. In the additional collinearity assessment, TyG-BMI, TyG-WC, and TyG-WHtR showed strong expected correlations because they share overlapping TyG-related metabolic and anthropometric components, as shown in Supplementary Tables 9 and 10. Therefore, the individual coefficients of these TyG-related adiposity indices should be interpreted cautiously, and the final combined model should be viewed primarily as a prediction model rather than an etiological model of independent biomarker effects.

The observed nonlinear relationships for ALI and NPAR with all-cause mortality also merit attention. These patterns are clinically plausible because composite inflammatory-nutritional markers may capture threshold-type risk, whereby worsening systemic inflammation or reduced nutritional reserve translates into disproportionately greater risk once a clinically meaningful range has been crossed [42–44]. NPAR combines neutrophil predominance and hypoalbuminemia and has been associated with adverse mortality outcomes in other population-based settings [21, 42]. ALI, originally developed as a prognostic composite of BMI, albumin, and neutrophil-to-lymphocyte ratio, likewise reflects the balance between inflammatory activation and physiological reserve [43, 44]. Taken together, the spline findings suggest that risk interpretation for these indices may be more informative when framed in clinically relevant ranges rather than as constant per-unit linear effects.

The TyG family offers a complementary interpretation centred on insulin resistance and adiposity-related vulnerability [45, 46]. TyG-based measures have been widely used as pragmatic surrogates of insulin resistance and cardiometabolic risk [12, 16, 17, 47, 48]. Modified TyG indices that incorporate anthropometric burden, such as TyG-BMI, TyG-WC, and TyG-WHtR, may better reflect the interaction between metabolic dysfunction and adiposity than TyG alone [49, 50]. In our study, however, TyG-related indices were less consistent than ALI across the adjusted association analyses, and their contribution appeared most useful when combined with inflammatory-nutritional information rather than interpreted in isolation. This pattern supports the idea that OSA-related mortality risk is unlikely to be adequately captured by a single metabolic axis.

From a prediction perspective, the continuous-scale combined model performed better than the tertile-based alternative and showed acceptable optimism-corrected discrimination, reasonable calibration, lower Brier score, and more favourable decision-curve characteristics. This finding is consistent with recent translational studies showing that integrated multi-marker signatures may improve risk stratification beyond single biomarkers [51–54]. This is clinically relevant because it moves the manuscript beyond a simple biomarker-ranking exercise toward a more pragmatic risk-benchmarking framework. Equally important, the model was compared against a prespecified base model rather than relying only on standalone AUC values. The finding that the combined biomarker model improved model fit and net reclassification, while producing only a modest increase in AUC, is more credible than an unrealistically large gain in discrimination and is consistent with the incremental manner in which biomarkers typically add value in clinical prediction.

A major strength of the present study is that the prediction framework was not only developed in a large survey-weighted NHANES cohort with questionnaire-defined OSA, but also externally validated in an independent multicenter Chinese cohort with PSG-confirmed OSA (AHI ≥ 5 events/hour) [27–29, 55]. Importantly, the externally validated object was the final Base + Combine model, and its performance was assessed alongside the base clinical model to maintain consistency with the derivation-stage incremental framework. All included patients in the external validation cohort had at least 5 years of observation time from baseline assessment, allowing the external endpoint to be defined as a fully observed binary 5-year all-cause mortality outcome rather than a censored time-to-event endpoint. A key methodological issue in the present study is the difference in OSA ascertainment between the derivation and external validation cohorts. The NHANES derivation cohort represented adults with questionnaire-defined probable OSA, whereas the external validation cohort consisted of patients with PSG-confirmed OSA. This difference introduced heterogeneity in diagnostic certainty, disease severity, referral patterns, baseline mortality risk, treatment exposure, and healthcare setting. Because PSG-derived severity metrics were unavailable in NHANES, the derivation cohort should be interpreted as a broad questionnaire-defined probable-OSA population rather than an objectively phenotyped sleep-laboratory cohort. This represents an important limitation, as it may have introduced disease misclassification and prevented adjustment for objective OSA severity measures, including AHI, oxygen desaturation index, and hypoxic burden. At the same time, this setting reflects large-scale epidemiologic, community-based, and lower-resource scenarios in which PSG-derived metrics are often unavailable and readily obtainable inflammatory, nutritional, and metabolic biomarkers may provide a low-cost entry point for pragmatic mortality risk benchmarking. The subsequent validation in a multicenter PSG-confirmed OSA cohort provided an opportunity to examine whether a biomarker-based risk-benchmarking model derived from a broad population-based probable-OSA setting could be transported to an objectively diagnosed clinical OSA population. This cross-setting validation is more informative than internal validation within NHANES alone, but it should also be interpreted cautiously. The case-definition and case-mix shift between the two cohorts may have reduced transportability and contributed to the modest external discrimination and poor calibration observed in the external cohort.

In the external cohort, the final Base + Combine model showed slightly higher discrimination than the base model, although calibration remained substantially poor for both models. In particular, the large calibration intercept, low calibration slope, and strong compression of predicted risks near zero suggest that the final Base + Combine model is not currently suitable for direct absolute risk estimation in the external population without recalibration or further adaptation. External decision-curve analysis further suggested that the incremental value of biomarker augmentation was threshold-dependent rather than uniform: the base model performed better at lower threshold probabilities, whereas the final Base + Combine model showed greater net benefit mainly at higher thresholds. Given that the observed 5-year mortality in the external cohort was 9%, this finding indicates that the practical clinical usefulness of the combined model may be narrower than a reader might infer from discrimination alone. Taken together, these findings should be regarded as exploratory evidence of transportability and modest incremental enrichment, but they do not support direct clinical applicability, absolute risk prediction, or use of the final model as a clearly superior prediction tool in PSG-confirmed OSA populations without further recalibration and validation.

The exploratory findings also require careful interpretation. For example, respiratory mortality analyses were based on only 13 RD deaths in the derivation cohort and therefore remain underpowered. Likewise, sex-stratified analyses may still be biologically interesting, but they should be viewed as hypothesis-generating rather than definitive. In particular, the suggestive female-predominant respiratory mortality signals for several TyG-related indices were not supported by significant interaction testing and should not be overinterpreted. These observations may motivate future work, but they do not currently justify strong claims about sex-specific pathways or respiratory risk stratification.

Several limitations should be acknowledged. First, the derivation cohort was based on questionnaire-defined probable OSA rather than PSG-confirmed OSA. Because PSG-derived severity metrics were unavailable in NHANES, this may have introduced disease misclassification and prevented adjustment for objective OSA severity measures, including AHI, oxygen desaturation index, and hypoxic burden. However, this setting also reflects large-scale epidemiologic, community-based, and lower-resource scenarios in which PSG-derived metrics are often unavailable, and is therefore consistent with the pragmatic objective of developing a low-cost biomarker-based risk-benchmarking framework. Second, sample attrition was substantial, as many otherwise eligible NHANES participants were excluded due to missing biomarker data, which may affect generalizability. Third, although the model was externally validated in an independent multicenter cohort with PSG-confirmed OSA, the derivation and validation cohorts differed in geography, case definition, case mix, and data structure. Because PSG-derived severity metrics were unavailable in NHANES, the derivation cohort should be interpreted as a questionnaire-defined probable-OSA population rather than an objectively phenotyped sleep-laboratory cohort. Nevertheless, validation in a PSG-confirmed clinical cohort provided an opportunity to examine whether a low-cost biomarker-based risk-benchmarking model derived from a broad population-based setting could be transported to an objectively diagnosed OSA population. However, this case-definition and case-mix shift may have contributed to the modest external discrimination and poor calibration, and therefore the external validation findings should be regarded as exploratory rather than definitive. Further validation and recalibration in larger PSG-confirmed cohorts are required before clinical implementation [29, 55]. In addition, substantial attrition occurred during assembly of the external validation cohort: 1023 PSG-confirmed patients were screened, but only 200 were included in the final analysis because of biomarker completeness requirements, availability of model covariates, and the need for fully observed 5-year outcome ascertainment. Although these exclusions were methodologically necessary, they may have introduced selection bias and altered the representativeness of the final external sample. This level of data completeness required for validation may also represent a practical barrier to implementation in routine clinical settings. Fourth, the external validation cohort included only 18 mortality events for evaluating a multivariable prediction model that incorporated routine clinical variables and a five-biomarker panel, which is below conventional events-per-variable considerations for stable model assessment and may have introduced substantial uncertainty into the estimated AUC of 0.697. More importantly, the poor external calibration indicates that the final Base + Combine model is not suitable for direct absolute risk estimation or clinical decision-making in new PSG-confirmed OSA populations without recalibration. In addition, we did not perform an exploratory recalibration-in-the-large analysis in the current manuscript. Therefore, we were unable to distinguish how much of the observed calibration error reflected baseline-risk shift versus deeper structural non-transportability. Fifth, objective OSA severity metrics such as AHI or hypoxic burden were not available in the NHANES derivation framework, so we were unable to test whether the biomarker panel added value beyond physiological severity measures [8–10]. Finally, biomarkers and covariates were measured only at baseline, and longitudinal changes or treatment effects, including CPAP use and cardiometabolic therapies, were not captured [56, 57].

Despite these limitations, the present study has several strengths and translational implications. It benchmarked seven routinely available inflammatory and metabolic indices head-to-head in probable OSA, developed a pragmatic risk-prediction framework in a large survey-weighted NHANES cohort, and externally validated the final Base + Combine model in an independent multicenter cohort with PSG-confirmed OSA. In addition, the analysis moved beyond biomarker ranking by incorporating calibration, Brier scores, decision curve analysis, and comparisons against a prespecified base model. From a translational perspective, these readily obtainable and low-cost biomarkers may be particularly useful in settings where PSG-based severity metrics are unavailable or difficult to implement. Although these biomarkers cannot replace PSG for diagnosis, they may still support pragmatic risk benchmarking and help identify patients who warrant closer follow-up in selected settings where PSG-derived severity metrics are unavailable. However, their broader clinical usefulness remains constrained by poor external calibration and by the relatively narrow threshold range in which incremental net benefit was observed. Future work should validate these findings in objectively phenotyped OSA cohorts, incorporate OSA severity metrics when available, recalibrate models across healthcare systems, and prospectively test whether biomarker-informed risk estimates can meaningfully improve clinical management.

## Conclusion

In adults with questionnaire-defined OSA, ALI showed the most robust mortality-related signal in this secondary NHANES analysis, while NPAR showed supportive signal in selected analyses. A combined inflammatory-metabolic biomarker model provided modest incremental enrichment beyond a prespecified base clinical model. These findings support a pragmatic biomarker-based approach to risk benchmarking in probable OSA, especially where PSG-derived severity metrics are unavailable. The cross-setting validation from a questionnaire-defined NHANES derivation cohort to a PSG-confirmed multicenter clinical cohort provides a scientifically informative, but more stringent, assessment of model transportability. However, the external validation results showed only modest discrimination and poor calibration, and should therefore be interpreted cautiously as preliminary rather than definitive. In particular, poor external calibration suggests that the final Base + Combine model is not yet suitable for direct absolute risk estimation in a new clinical population without further recalibration or refinement. Further validation in larger objectively phenotyped cohorts is warranted before broader clinical decision support can be considered.

## Electronic supplementary material

Below is the link to the electronic supplementary material.

Supplementary Material 1

Supplementary Material 2

Supplementary Material 3

Supplementary Material 4

Supplementary Material 5

Supplementary Material 6

Supplementary Material 7

Supplementary Material 8

Supplementary Material 9

Supplementary Material 10

Supplementary Material 11

## Data Availability

The NHANES datasets and documentation used for the derivation cohort are publicly available from the Centers for Disease Control and Prevention (CDC)/National Center for Health Statistics (NCHS) NHANES website. The NHANES-linked mortality data (National Death Index linkage through December 31, 2019) are also available through NCHS. The multicenter Chinese external validation dataset is not publicly available because it contains clinical data subject to ethics and privacy restrictions. Additional details are available from the corresponding author upon reasonable request.

## Abbreviations

AHI: apnea–hypopnea index
ALI: advanced lung cancer inflammation index
AUC: area under the curve
BMI: body mass index
CDC: Centers for Disease Control and Prevention
CI: confidence interval
CPAP: continuous positive airway pressure
CVD: cardiovascular disease
DCA: decision-curve analysis
HDL-C: high-density lipoprotein cholesterol
HR: hazard ratio
IQR: interquartile range
NCHS: National Center for Health Statistics
NDI: National Death Index
NHANES: National Health and Nutrition Examination Survey
NLR: neutrophil-to-lymphocyte ratio
NPAR: neutrophil percentage-to-albumin ratio
NRI: net reclassification improvement
OSA: obstructive sleep apnea
OR: odds ratio
PIR: poverty income ratio
PSG: polysomnography
RCS: restricted cubic spline
RD: respiratory disease
ROC: receiver operating characteristic
SD: standard deviation
TG: triglycerides
TG/HDL-C: triglyceride-to-high-density lipoprotein cholesterol ratio
TyG: triglyceride–glucose index
TyG-BMI: triglyceride–glucose index multiplied by body mass index
TyG-WC: triglyceride–glucose index multiplied by waist circumference
TyG-WHtR: triglyceride–glucose index multiplied by waist-to-height ratio
WC: waist circumference
WHtR: waist-to-height ratio
