# Supplementary material for: Benchmarking inflammation-nutrition and TyG-related indices for 5-year mortality risk in adults with questionnaire-defined obstructive sleep apnea: a survey-weighted NHANES derivation cohort with multicenter external validation
Source: J Transl Med. 2026 Jul 10;24:901. doi: 10.1186/s12967-026-08540-0 (PMC13366672; doi:10.1186/s12967-026-08540-0)
Supplement: Supplementary file 1 — Supplementary Material 1 [file 12967_2026_8540_MOESM1_ESM.pdf]

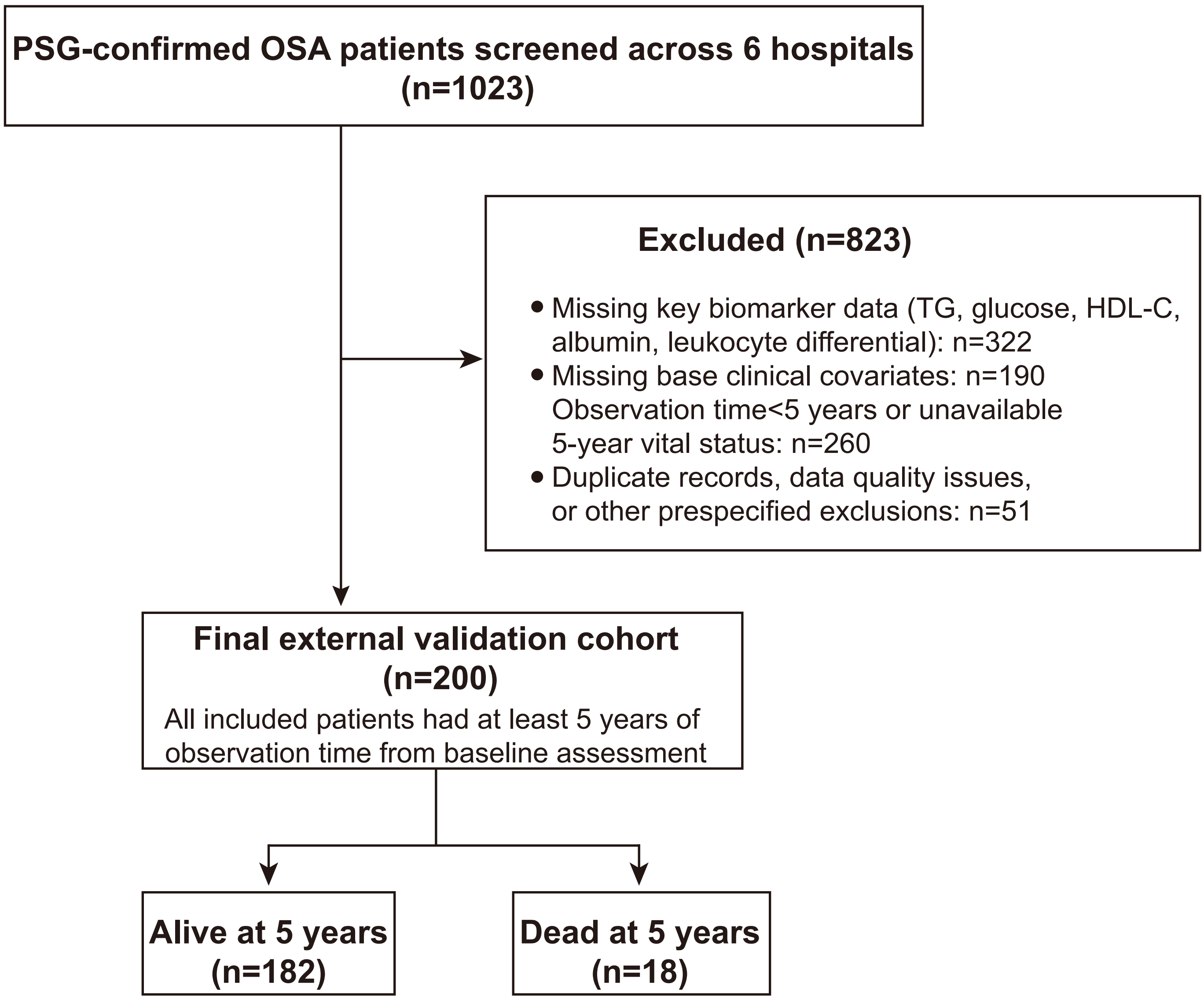
PSG-confirmed OSA patients screened across 6 hospitals (n=1023)
Excluded (n=823)
Missing key biomarker data (TG, glucose, HDL-C, albumin, leukocyte differential): n=322
Missing base clinical covariates: n=190
Observation time<5 years or unavailable 5-year vital status: n=260
Duplicate records, data quality issues, or other prespecified exclusions: n=51
Final external validation cohort (n=200)
All included patients had at least 5 years of observation time from baseline assessment
Alive at 5 years (n=182)
Dead at 5 years (n=18)
