# Supplementary figures and images for: Benchmarking inflammation-nutrition and TyG-related indices for 5-year mortality risk in adults with questionnaire-defined obstructive sleep apnea: a survey-weighted NHANES derivation cohort with multicenter external validation
Source: J Transl Med. 2026 Jul 10;24:901. doi: 10.1186/s12967-026-08540-0 (PMC13366672; doi:10.1186/s12967-026-08540-0)

A

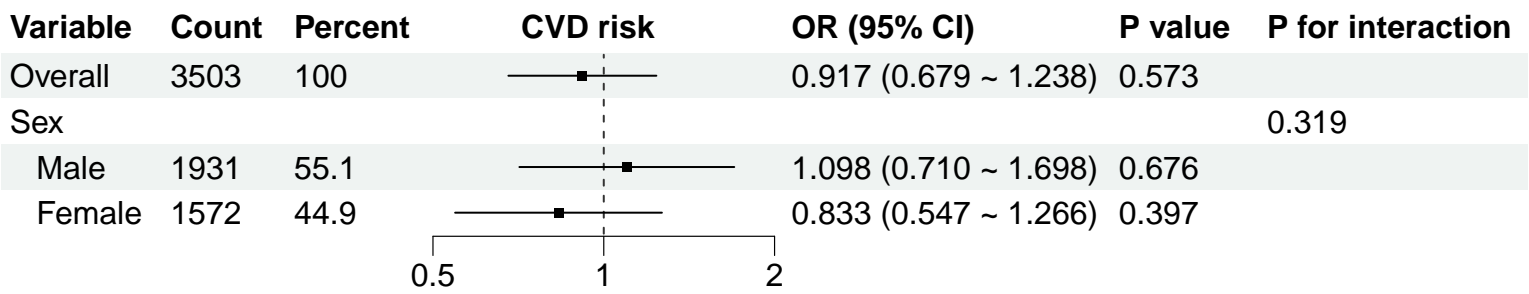

B

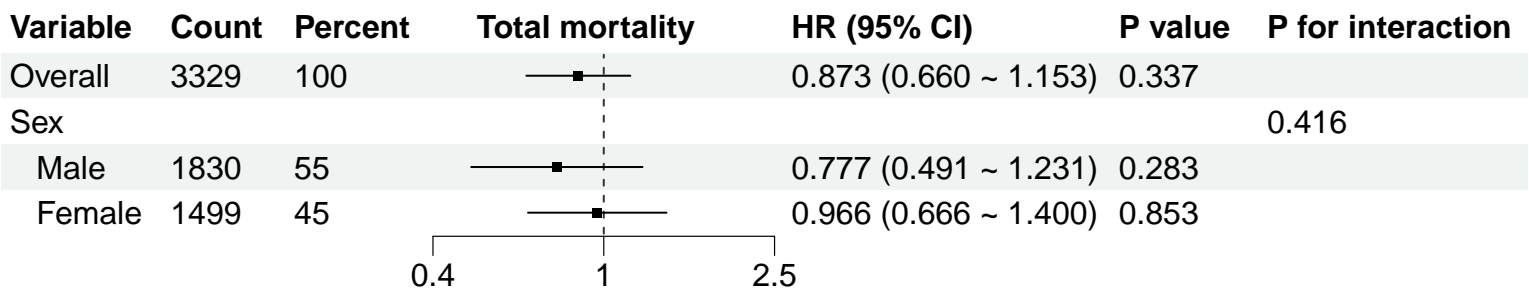

C

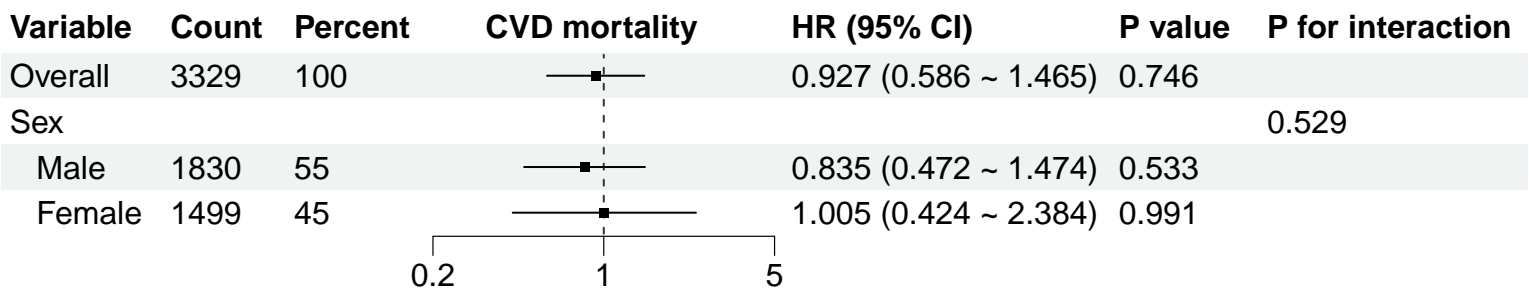

D

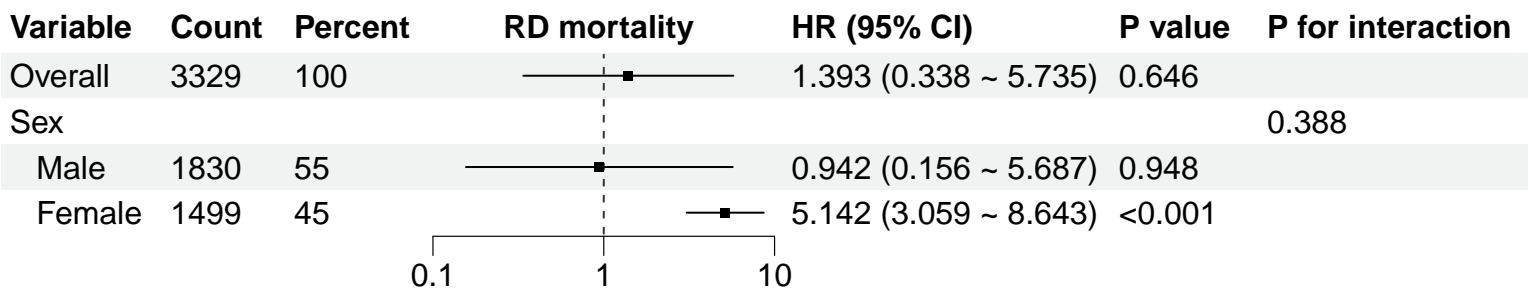

Supplement: Supplementary file 2 — Supplementary Material 2 [file 12967_2026_8540_MOESM2_ESM.pdf]

A

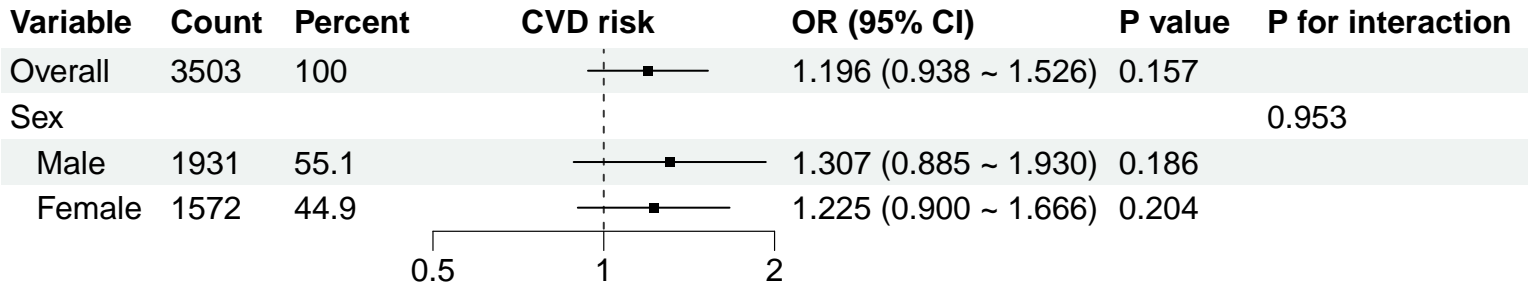

B

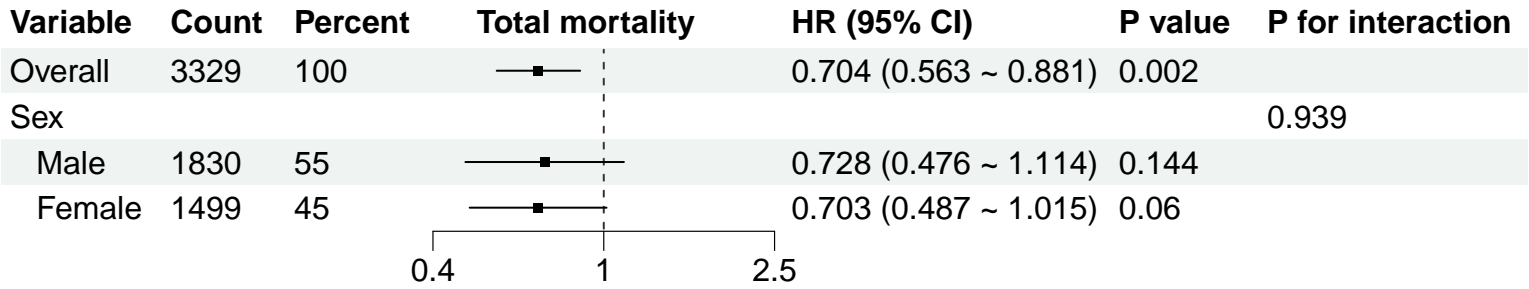

C

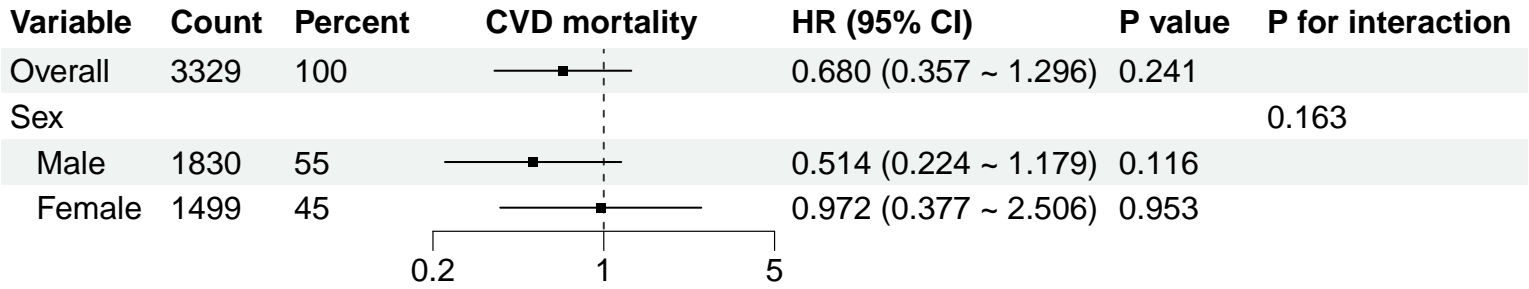

D

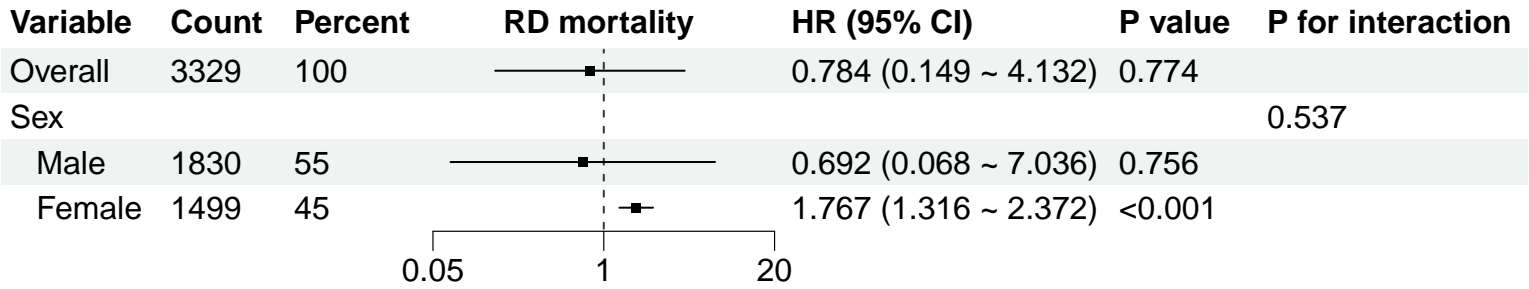

Supplement: Supplementary file 3 — Supplementary Material 3 [file 12967_2026_8540_MOESM3_ESM.pdf]

A

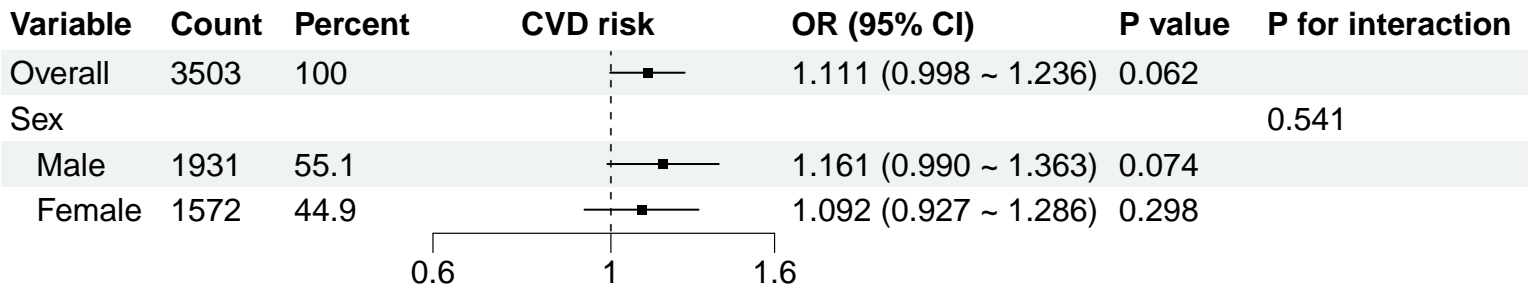

B

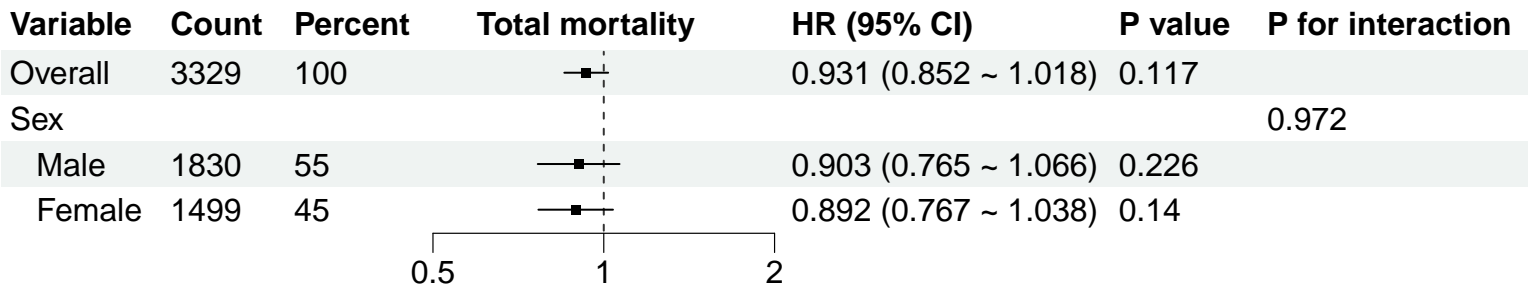

C

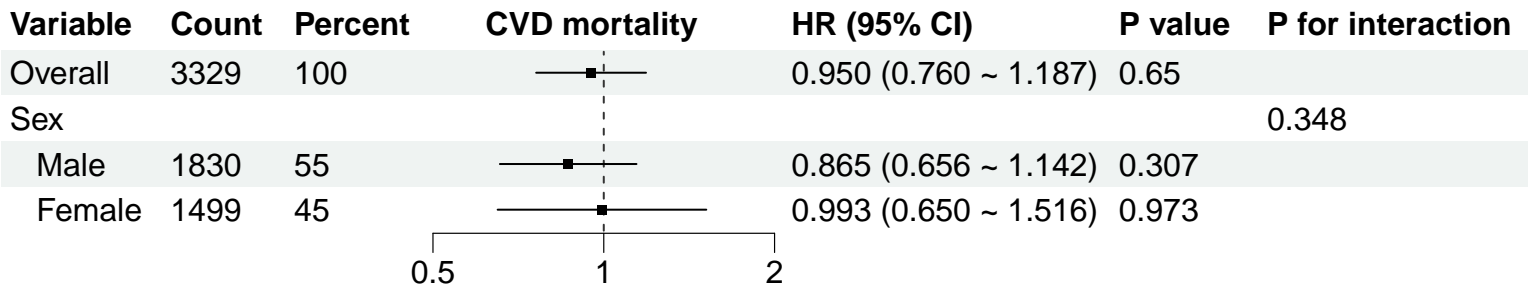

D

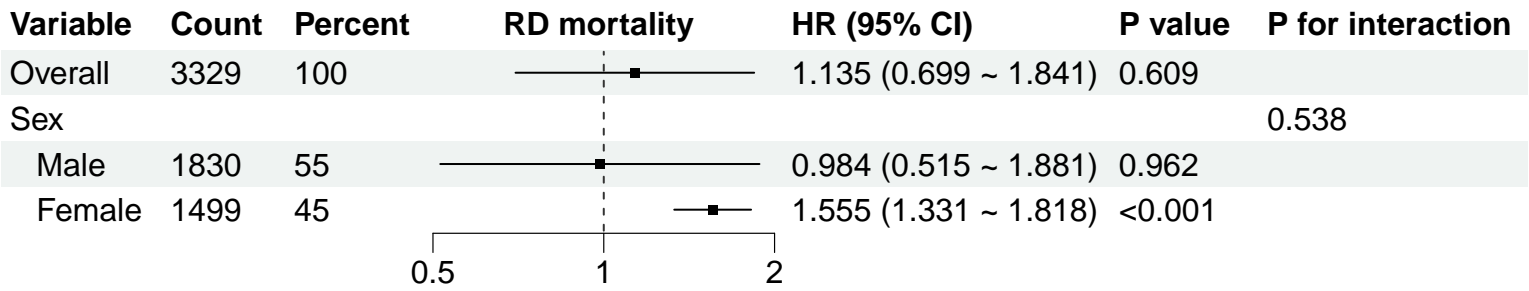

Supplement: Supplementary file 4 — Supplementary Material 4 [file 12967_2026_8540_MOESM4_ESM.pdf]

A

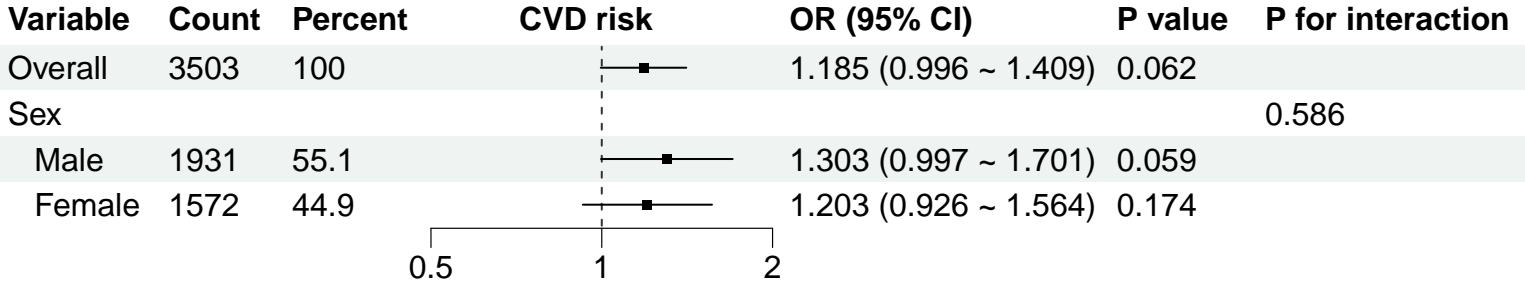

B

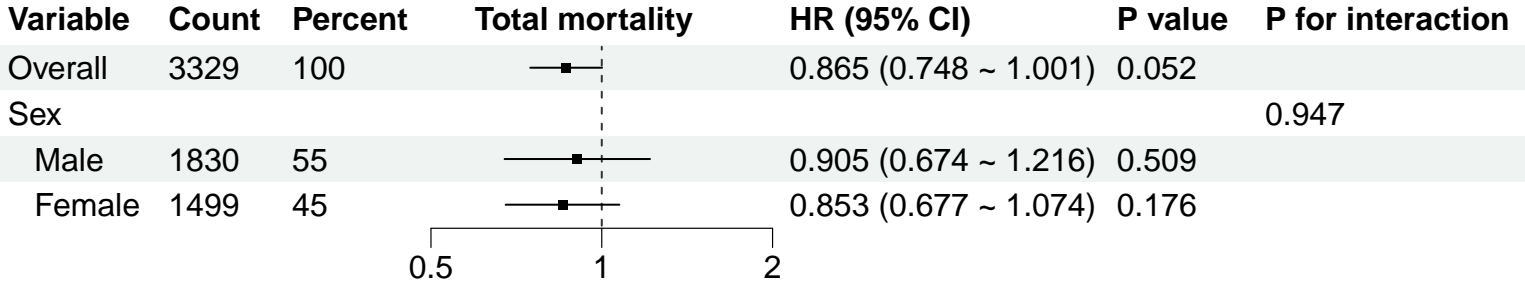

C

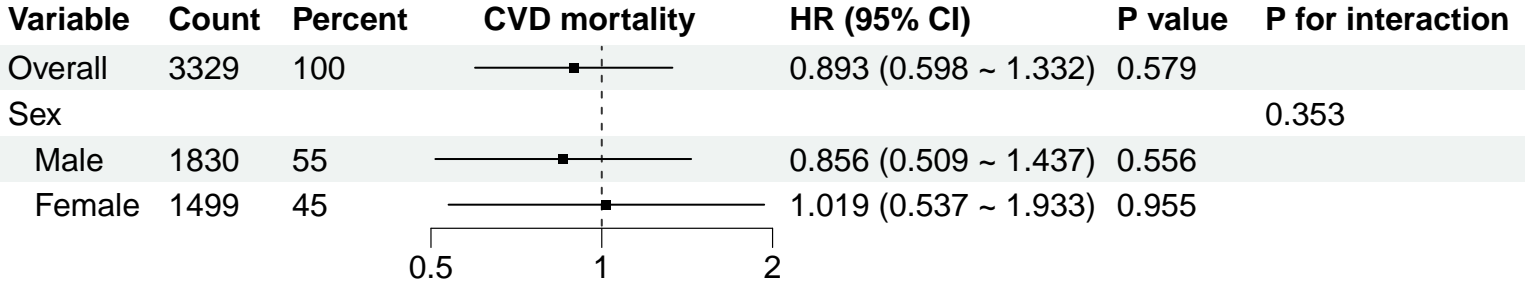

D

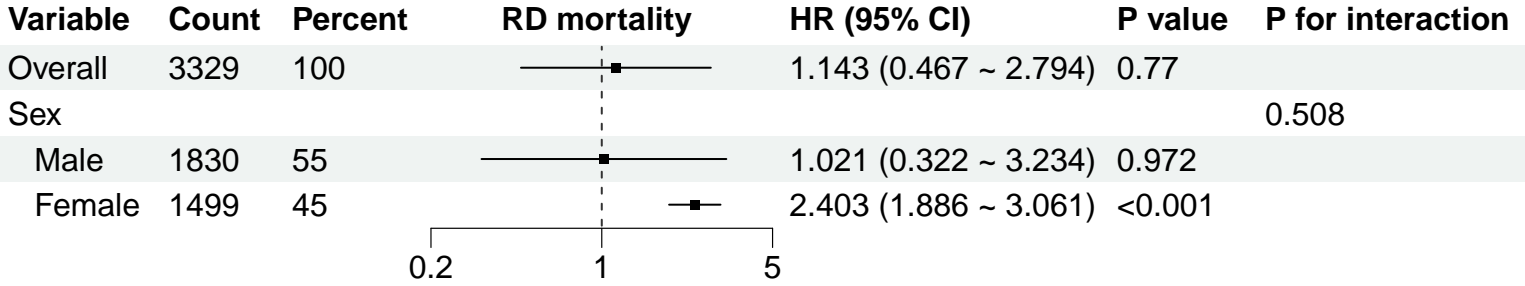

Supplement: Supplementary file 5 — Supplementary Material 5 [file 12967_2026_8540_MOESM5_ESM.pdf]

A

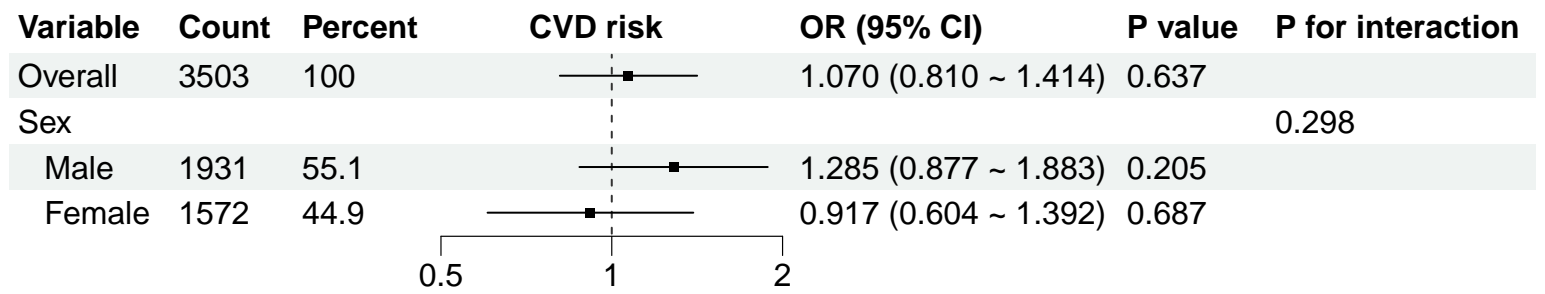

B

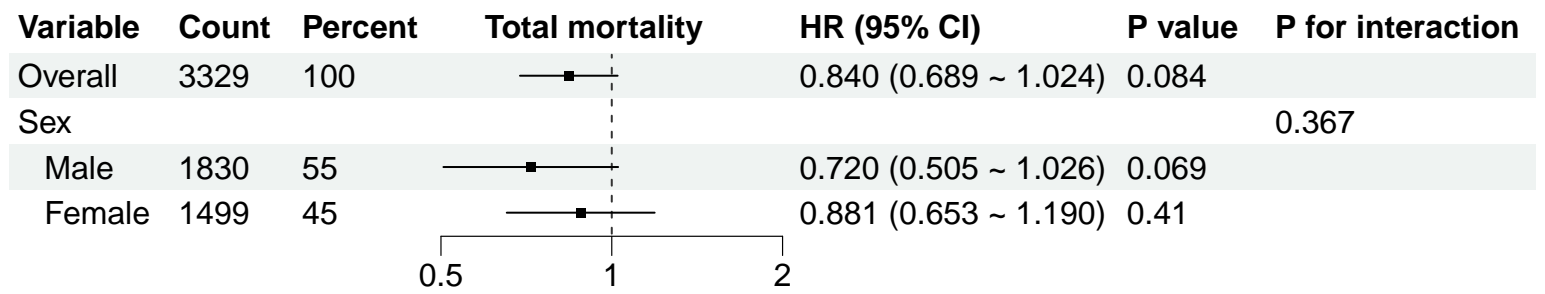

C

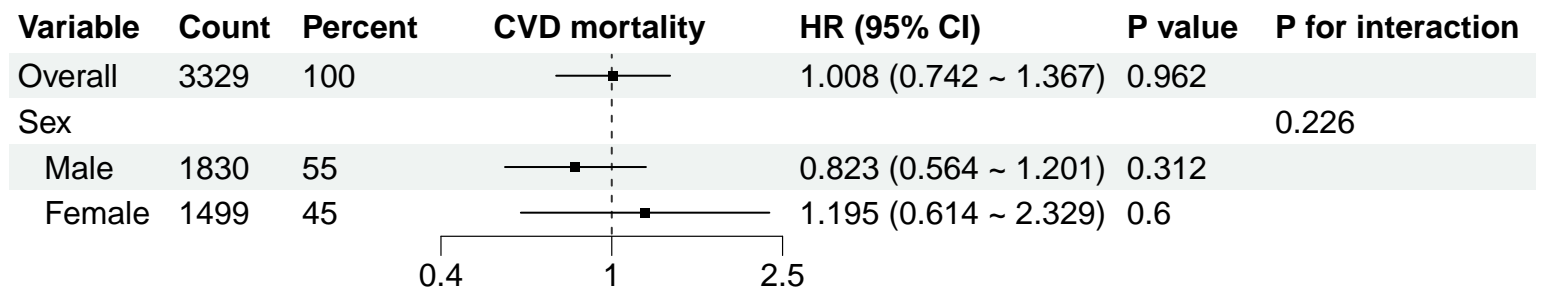

D

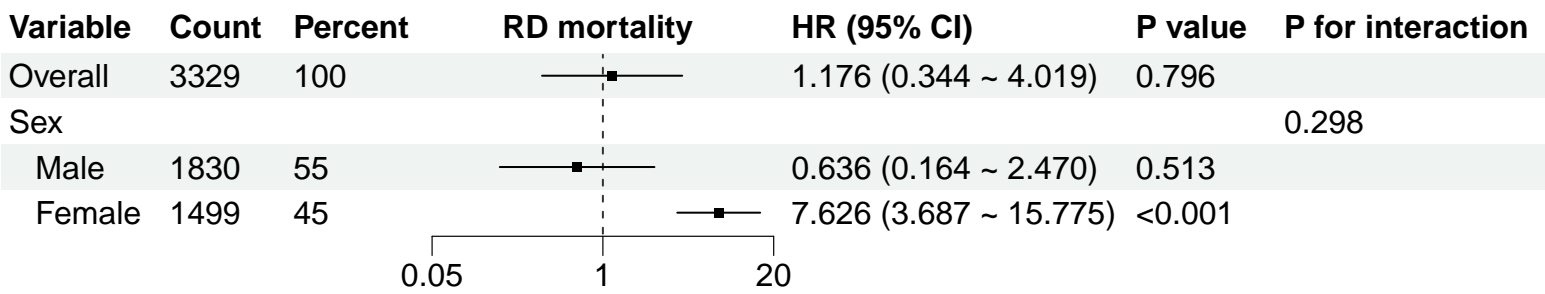

Supplement: Supplementary file 6 — Supplementary Material 6 [file 12967_2026_8540_MOESM6_ESM.pdf]

A

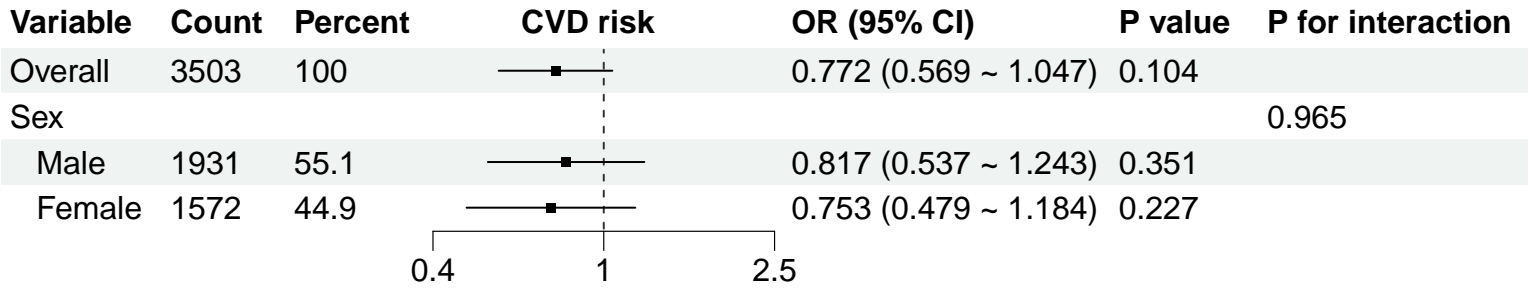

B

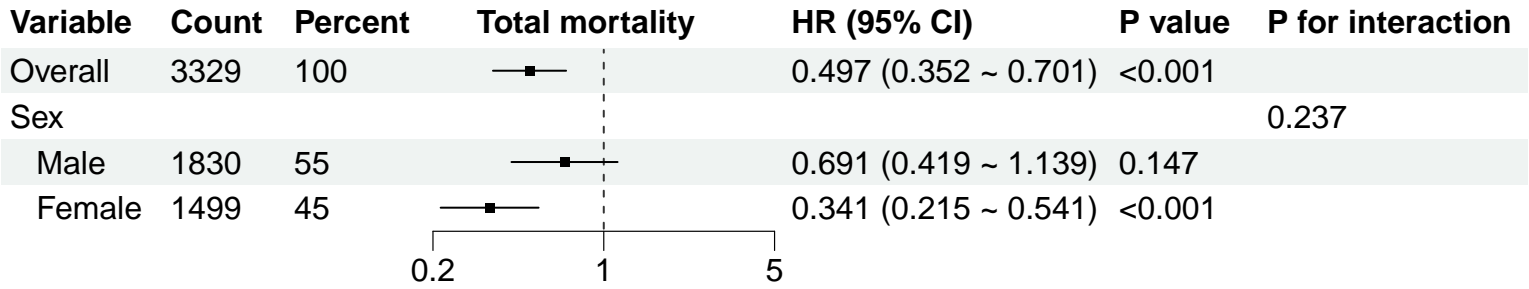

C

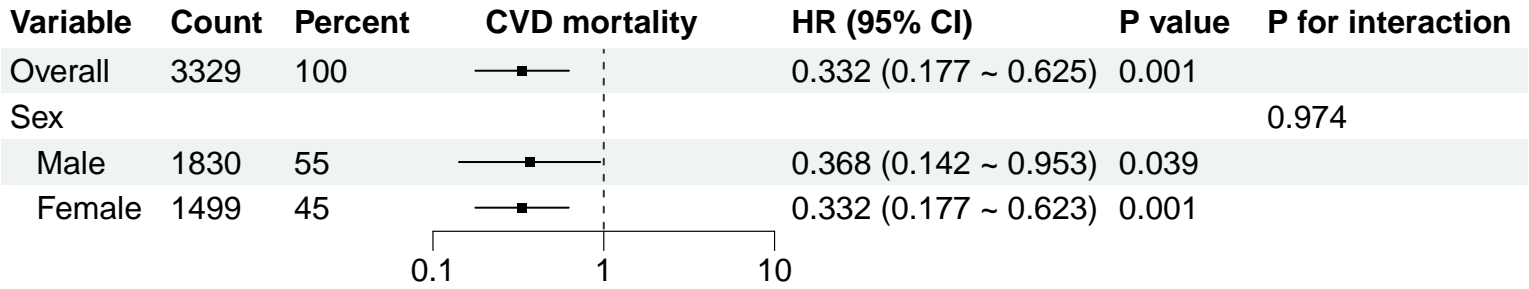

D

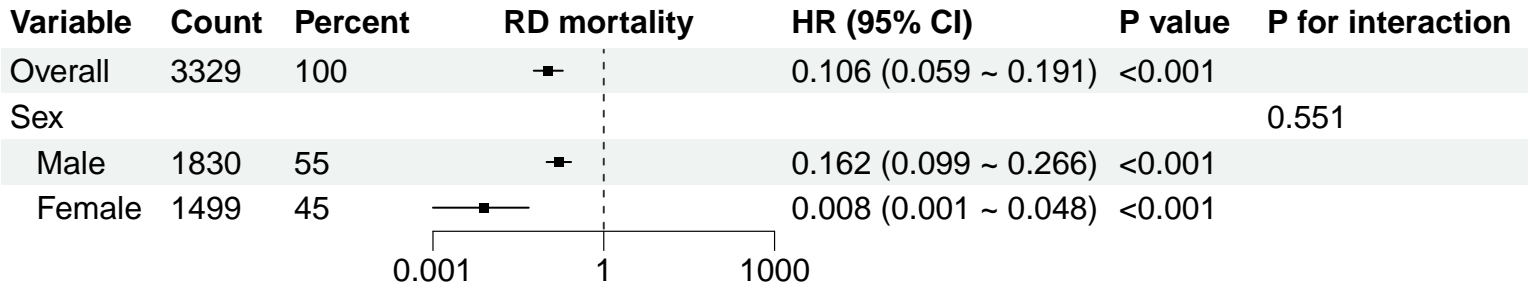

Supplement: Supplementary file 7 — Supplementary Material 7 [file 12967_2026_8540_MOESM7_ESM.pdf]

A

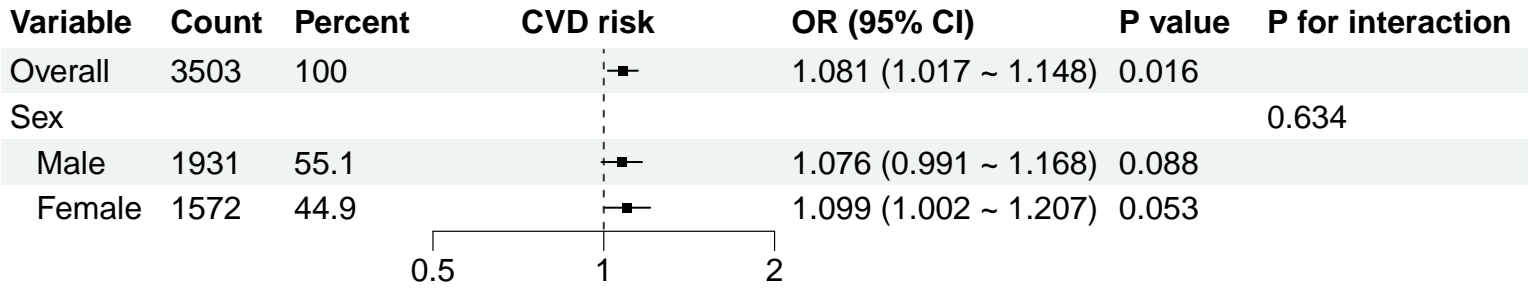

B

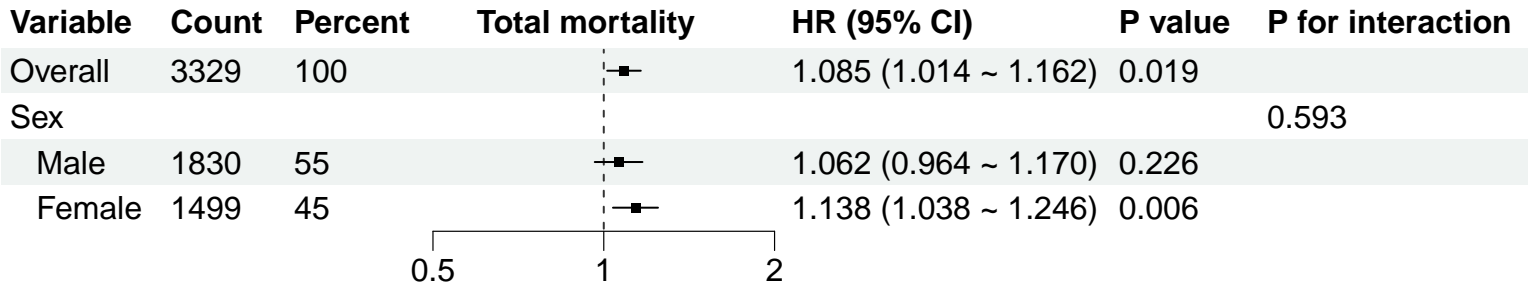

C

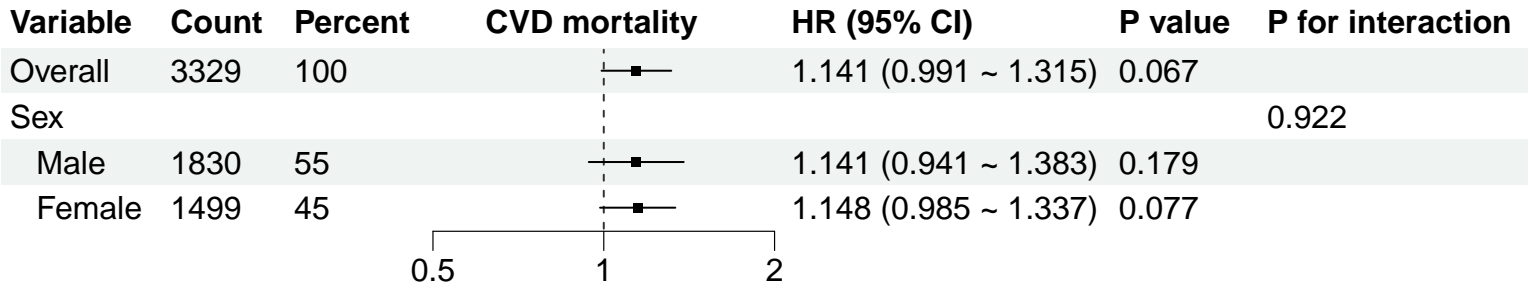

D

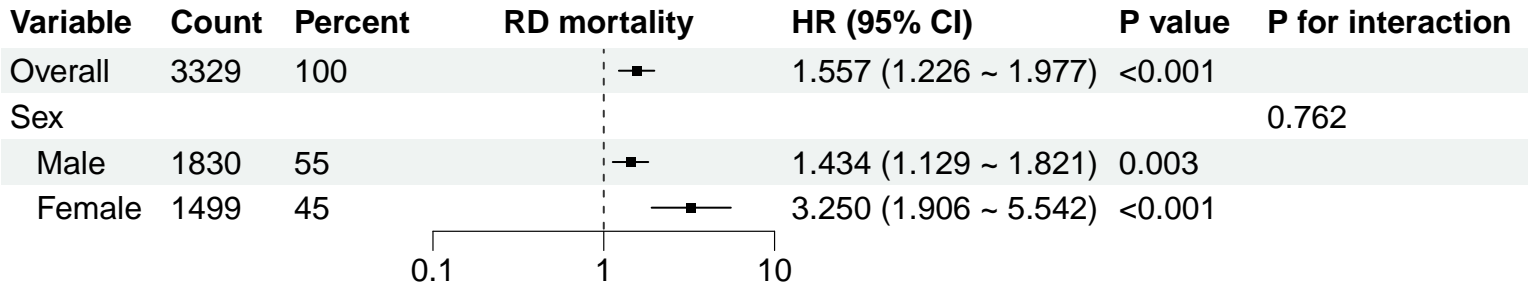

Supplement: Supplementary file 8 — Supplementary Material 8 [file 12967_2026_8540_MOESM8_ESM.pdf]
