# Supplementary material for: Benchmarking inflammation-nutrition and TyG-related indices for 5-year mortality risk in adults with questionnaire-defined obstructive sleep apnea: a survey-weighted NHANES derivation cohort with multicenter external validation
Source: J Transl Med. 2026 Jul 10;24:901. doi: 10.1186/s12967-026-08540-0 (PMC13366672; doi:10.1186/s12967-026-08540-0)
Supplement: Supplementary file 9 — Supplementary Material 9 [file 12967_2026_8540_MOESM9_ESM.docx]

**Supplementary Methods. Exact linear predictor formulas and worked example of risk calculation**

**Exact linear predictor formulas**

For the base clinical model, the linear predictor was defined as:

η_base = 1.1347 × I(Age 45–<60) + 2.3795 × I(Age ≥60) − 0.3454 × I(Female)
− 0.4197 × I(Overweight) − 0.5467 × I(Obesity) + 1.0362 × I(Underweight)

- 0.3625 × I(Diabetes) + 0.5513 × I(Hypertension)
- 0.5874 × I(Ever smoker) + 0.8819 × I(Current smoker)
  − 0.0819 × I(Ever drinking) − 0.4135 × I(Mild drinking)
  − 0.8694 × I(Moderate drinking) − 0.3914 × I(Heavy drinking)

For the final Base + Combine model, the linear predictor was defined as:

η_combine = −1.1320 × TyGBMI100 + 0.2881 × TyGWC100 + 0.4781 × TyGWHtR
− 0.4105 × ln(TG/HDL-C) − 0.7340 × ln(ALI)

- 0.9793 × I(Age 45–<60) + 2.0248 × I(Age ≥60) − 0.3074 × I(Female)
  − 0.3355 × I(Overweight) − 0.3533 × I(Obesity) + 1.2273 × I(Underweight)
- 0.2531 × I(Diabetes) + 0.5001 × I(Hypertension)
- 0.5329 × I(Ever smoker) + 0.8092 × I(Current smoker)
  − 0.0160 × I(Ever drinking) − 0.4374 × I(Mild drinking)
  − 0.8160 × I(Moderate drinking) − 0.3839 × I(Heavy drinking)

Reference categories were age <45 years, male sex, normal BMI, no diabetes, no hypertension, never smoking, and never drinking. TyGBMI100 and TyGWC100 denote TyG-BMI and TyG-WC divided by 100, respectively, and ln() denotes the natural logarithm.

For both models, the relative hazard was calculated as HR = exp(η). If absolute 5-year risk is required from the Cox model, it may be derived as:

Risk (5 years) = 1 − S0(5)^exp(η)

where S0(5) denotes the model-specific 5-year baseline survival.

**Worked example of risk calculation**

As an example, consider a hypothetical patient with the following characteristics: age ≥60 years, female sex, overweight, diabetes, hypertension, ever smoker, mild drinking, TyG-BMI = 260, TyG-WC = 910, TyG-WHtR = 5.60, TG/HDL-C = 1.50, and ALI = 55.

For this patient:

TyGBMI100 = 2.60

TyGWC100 = 9.10

ln(TG/HDL-C) = ln(1.50)

ln(ALI) = ln(55)

Substituting these values into the final Base + Combine model gives:

η_combine = −1.1320 × 2.60 + 0.2881 × 9.10 + 0.4781 × 5.60
− 0.4105 × ln(1.50) − 0.7340 × ln(55)

- 2.0248 − 0.3074 − 0.3355 + 0.2531 + 0.5001 + 0.5329 − 0.4374
  = 1.479

Thus, the relative hazard is:

HR = exp(1.479) = 4.39

If the model-specific 5-year baseline survival S0(5) is available, the corresponding absolute 5-year risk may be calculated as:

Risk (5 years) = 1 − S0(5)^4.39

This worked example is provided for illustration of model use only. Absolute 5-year risk estimation additionally requires the model-specific 5-year baseline survival, S0(5).
