# Supplementary material for: Benchmarking inflammation-nutrition and TyG-related indices for 5-year mortality risk in adults with questionnaire-defined obstructive sleep apnea: a survey-weighted NHANES derivation cohort with multicenter external validation
Source: J Transl Med. 2026 Jul 10;24:901. doi: 10.1186/s12967-026-08540-0 (PMC13366672; doi:10.1186/s12967-026-08540-0)
Supplement: Supplementary file 11 — Supplementary Material 11 [file 12967_2026_8540_MOESM11_ESM.docx]

**Supplementary Figure Legend**s

Supplementary Figure 1. Flow diagram of the external validation cohort.

Supplementary Figure 2. Sex-stratified continuous-scale associations of TyG with clinical outcomes.

Forest plots showing sex-stratified associations of TyG, modelled as a continuous variable, with (A) prevalent cardiovascular disease (CVD), (B) all-cause mortality, (C) cardiovascular mortality, and (D) respiratory disease (RD) mortality. Estimates are shown for the overall cohort and separately for men and women, together with the P value for interaction.

Supplementary Figure 3. Sex-stratified continuous-scale associations of TyG-BMI with clinical outcomes.

Forest plots showing sex-stratified associations of TyG-BMI, modelled on the continuous scale, with (A) prevalent CVD, (B) all-cause mortality, (C) cardiovascular mortality, and (D) RD mortality. Estimates are shown for the overall cohort and separately for men and women, together with the P value for interaction.

Supplementary Figure 4. Sex-stratified continuous-scale associations of TyG-WC with clinical outcomes.

Forest plots showing sex-stratified associations of TyG-WC, modelled on the continuous scale, with (A) prevalent CVD, (B) all-cause mortality, (C) cardiovascular mortality, and (D) RD mortality. Estimates are shown for the overall cohort and separately for men and women, together with the P value for interaction.

Supplementary Figure 5. Sex-stratified continuous-scale associations of TyG-WHtR with clinical outcomes.

Forest plots showing sex-stratified associations of TyG-WHtR, modelled on the continuous scale, with (A) prevalent CVD, (B) all-cause mortality, (C) cardiovascular mortality, and (D) RD mortality. Estimates are shown for the overall cohort and separately for men and women, together with the P value for interaction.

Supplementary Figure 6. Sex-stratified continuous-scale associations of TG/HDL-C with clinical outcomes.

Forest plots showing sex-stratified associations of TG/HDL-C, modelled on the continuous scale, with (A) prevalent CVD, (B) all-cause mortality, (C) cardiovascular mortality, and (D) RD mortality. Estimates are shown for the overall cohort and separately for men and women, together with the P value for interaction.

Supplementary Figure 7. Sex-stratified continuous-scale associations of ALI with clinical outcomes.

Forest plots showing sex-stratified associations of ALI, modelled on the continuous scale, with (A) prevalent CVD, (B) all-cause mortality, (C) cardiovascular mortality, and (D) RD mortality. Estimates are shown for the overall cohort and separately for men and women, together with the P value for interaction.

Supplementary Figure 8. Sex-stratified continuous-scale associations of NPAR with clinical outcomes.

Forest plots showing sex-stratified associations of NPAR, modelled on the continuous scale, with (A) prevalent CVD, (B) all-cause mortality, (C) cardiovascular mortality, and (D) RD mortality. Estimates are shown for the overall cohort and separately for men and women, together with the P value for interaction.

Supplementary Table 9. Spearman correlation matrix of biomarkers included in Model 2

Supplementary Table 10. Variance inflation factor analysis of biomarkers included in Model 2

**Supplementary Table legends**

Supplementary Table 1. Regression coefficients of the base model and the final Base + Combine model for 5-year all-cause mortality prediction in adults with questionnaire-defined OSA in NHANES.

Supplementary Table 2. Unweighted baseline characteristics of adults with questionnaire-defined obstructive sleep apnea (OSA) in the NHANES derivation cohort, stratified by prevalent cardiovascular disease (CVD), all-cause mortality, cardiovascular mortality, and respiratory disease (RD) mortality.

Supplementary Table 3. Survey-weighted baseline characteristics of adults with questionnaire-defined OSA in the NHANES derivation cohort, stratified by prevalent CVD, all-cause mortality, cardiovascular mortality, and RD mortality.

Supplementary Table 4. Comparison of baseline demographic and clinical characteristics between the NHANES derivation cohort and the independent external validation cohort with polysomnography-confirmed OSA from six hospitals in China.

Supplementary Table 5 OR (95% CIs) of CVD prevalence according to tertiles of seven biomarkers among OSA in NHANES.

Supplementary Table 6 HR (95% CIs) of CVD mortality according to tertiles of seven biomarkers among OSA in NHANES.

Supplementary Table 7 HR (95% CIs) of Respiratory disease mortality according to tertiles of seven biomarkers among OSA in NHANES.

Supplementary Table 8. Center-level descriptive characteristics of the external validation cohort

Supplementary Table 9. Spearman correlations of Model 2 biomarkers

Supplementary Table 10. VIF analysis of Model 2 biomarkers
